# αα-hub coregulator structure and flexibility determine transcription factor binding and selection in regulatory interactomes

**DOI:** 10.1016/j.jbc.2022.101963

**Published:** 2022-04-20

**Authors:** Frederik Friis Theisen, Edoardo Salladini, Rikke Davidsen, Christina Jo Rasmussen, Lasse Staby, Birthe B. Kragelund, Karen Skriver

**Affiliations:** 1 REPIN and the Linderstrøm-Lang Centre for Protein Science, Department of Biology, University of Copenhagen, Copenhagen, Denmark; 2 Structural Biology and NMR Laboratory, Department of Biology, University of Copenhagen, Copenhagen, Denmark

**Keywords:** αα-hub specificity, transcriptional coactivator, interactome size, alternative state, malleability, entropy, protein dynamic, protein structure, protein–protein interaction, intrinsically disordered protein, AD, activation domain, ANAC, *A. thaliana* NAM, ATAF1/2, and CUC2, *At*RCD1, *Arabidopsis thaliana* RCD1, CSP, chemical shift perturbation, DREB2A, dehydration-responsive element–binding protein 2A, DSS, 2,2-dimethyl-2-silapentane-5-sulphonate, *Hs*, *Homo sapiens*, HSQC, heteronuclear single quantum coherence, ITC, isothermal titration calorimetry, RCD1, radical-induced cell death1, RST, RCD1, SRO, and TAF4, SAXS, small-angle X-ray scattering, SLiM, short linear motif, SRO, similar to RCD one, TAF4, transcription initiation factor TFIID-subunit 4, TAFH, TATA-box–associated factor homology, TF, transcription factor

## Abstract

Formation of transcription factor (TF)–coregulator complexes is a key step in transcriptional regulation, with coregulators having essential functions as hub nodes in molecular networks. How specificity and selectivity are maintained in these nodes remain open questions. In this work, we addressed specificity in transcriptional networks using complexes formed between TFs and αα-hubs, which are defined by a common αα-hairpin secondary structure motif, as a model. Using NMR spectroscopy and binding thermodynamics, we analyzed the structure, dynamics, stability, and ligand-binding properties of the *Arabidopsis thaliana* RST domains from TAF4 and known binding partner RCD1, and the TAFH domain from human TAF4, allowing comparison across species, functions, and architectural contexts. While these αα-hubs shared the αα-hairpin motif, they differed in length and orientation of accessory helices as well as in their thermodynamic profiles of ligand binding. Whereas biologically relevant RCD1–ligand pairs displayed high affinity driven by enthalpy, TAF4–ligand interactions were entropy driven and exhibited less binding-induced structuring. We in addition identified a thermal unfolding state with a structured core for all three domains, although the temperature sensitivity differed. Thermal stability studies suggested that initial unfolding of the RCD1–RST domain localized around helix 1, lending this region structural malleability, while effects in TAF4–RST were more stochastic, suggesting variability in structural adaptability upon binding. Collectively, our results support a model in which hub structure, flexibility, and binding thermodynamics contribute to αα-hub–TF binding specificity, a finding of general relevance to the understanding of coregulator–ligand interactions and interactome sizes.

Signaling pathways, implicated in diverse biological processes such as stress responses and development, culminate in regulation of gene expression. For this, interactions between transcription factors (TFs) and coregulators are essential by guiding the transcriptional machinery to target genes (1, 2). Generally, activation domains (ADs) of TFs can bind multiple unrelated coregulators and vice versa (3, 4), and functionally, the ADs are interchangeable (5). ADs are not conserved at the sequence level (6), and structurally, they are often intrinsically disordered, lacking a defined folded structure (7, 8). Therefore, the interactions between ADs and coregulators have been regarded nonspecific with stochastic burial of hydrophobic residues and lack of long-lived intermolecular contacts (7, 9, 10, 11). As a result, multiple conformations and orientations of TF–coregulator complexes exist (9). However, recent studies revealed new principles of affinity and specificity for such complexes. For the large Gcn4–Med15 TF–coactivator complex, multiple domains contribute to affinity (10), and for the interactions between Ets TFs and Med25, even small sequence differences in the TFs affect specificity through conformational effects on Med25 (12). Thus, despite intensive studies for more than 30 years (7, 10, 12), TF–coregulator specificity remains enigmatic, and additional model systems are needed. One recently established model system is constituted by the αα-hub–TF interactions (13, 14). In this model system, topologically similar, evolutionary unrelated, αα-hub domains found throughout eukaryotes interact with numerous unrelated intrinsically disordered TFs using diverse molecular features.

The αα-hubs were recently defined based on structural and functional similarities of RST (radical-induced cell death1 [RCD1], similar to RCD one [SRO], and transcription initiation factor TFIID-subunit [TAF4]), paired amphipathic helix, TATA-box–associated factor homology (TAFH), harmonin–homology domain, and nuclear coactivator–binding domain of the important transcriptional regulators RCD1, Sin3, TAF4, and CREB-binding protein (13, 14, 15). αα-hubs are small (<100 residues) α-helical domains present in larger multidomain proteins, and they share an αα-hairpin super secondary motif, linking variable, malleable helices of different lengths. The prototypical αα-hub domain consists of four α-helices, and its αα-hairpin is stabilized by a hydrophobic β3-loop residue (13, 14, 15). Most αα-hub–containing proteins organize large interactomes (8, 13, 16, 17), with intrinsically disordered TFs being over-represented among αα-hub ligands (13) and thus typically act as coregulators of transcription.

RCD1 is a member of the plant-specific SRO family and contains several domains, one of which is the RST αα-hub domain (18). *Arabidopsis thaliana* RCD1 (*At*RCD1) plays important roles in stress responses and development (19, 20, 21), and in accordance with RCD1 functioning as a cellular hub protein (22), *rcd1* knockout mutants display pleiotropic effects in stress responses and development (19). RCD1 negatively affects abiotic stress responses *via* RST-mediated interactions with the TF dehydration-responsive element–binding protein 2A (DREB2A) (23) and *A. thaliana* NAM, ATAF1/2, and CUC2 (ANAC) 013 and ANAC017 (24, 25). Biochemically, *At*RCD1–RST is well characterized, and its NMR structure has been solved alone and in complex with DREB2A (14, 24), and the RCD1-binding short linear motif (SLiM) has been identified (26, 27, 28). The RST domain is also found in the plant paralogs TAF4 and TAF4b (18, 19, 29), encoded by genes with constitutive and narrow expression patterns, respectively (30). TAF4s are crucial for the structural integrity of the TFIID general TF complex (31, 32, 33). Based on the common architecture of *Arabidopsis* and human (*Homo sapiens* [*Hs*]) TAF4, their αα-hub domains, RST and TAFH, respectively, are likely to share the molecular function of interacting with TFs (13, 34, 35).

In this study, we addressed specificity in transcriptional networks using αα-hub–TF interactions as model invoking three different αα-hubs from three different hub proteins. Based on a comparison of their three-dimensional structures, one determined in this work, conformational stability, and binding thermodynamics, discrimination between ligands was apparent. Thus, high-affinity *At*RCD1–RST–TF interactions were driven by binding enthalpy, and lower affinity TAF4-αα-hub–TF interactions were driven by entropy. This discrimination was also manifested in different degrees of folding upon binding and likely reflects specific association of biologically relevant αα-hub–TF pairs and unspecific dynamic association of “random” ligands with the TAF4 αα-hub domains. A thermal unfolding state with a substantial helical core was identified for all three domains, but with different temperature sensitivity, suggesting variability in structural adaptability relevant to binding. Together, the results revealed that αα-hub–TF interactions depend not only on coupled folding and binding of both partners but also on the formation of specific contacts, which will facilitate maximum folding, a key factor toward specificity.

## Results

### Domain architectures and sequences may hold clues to interactomes

**Figure 1:**
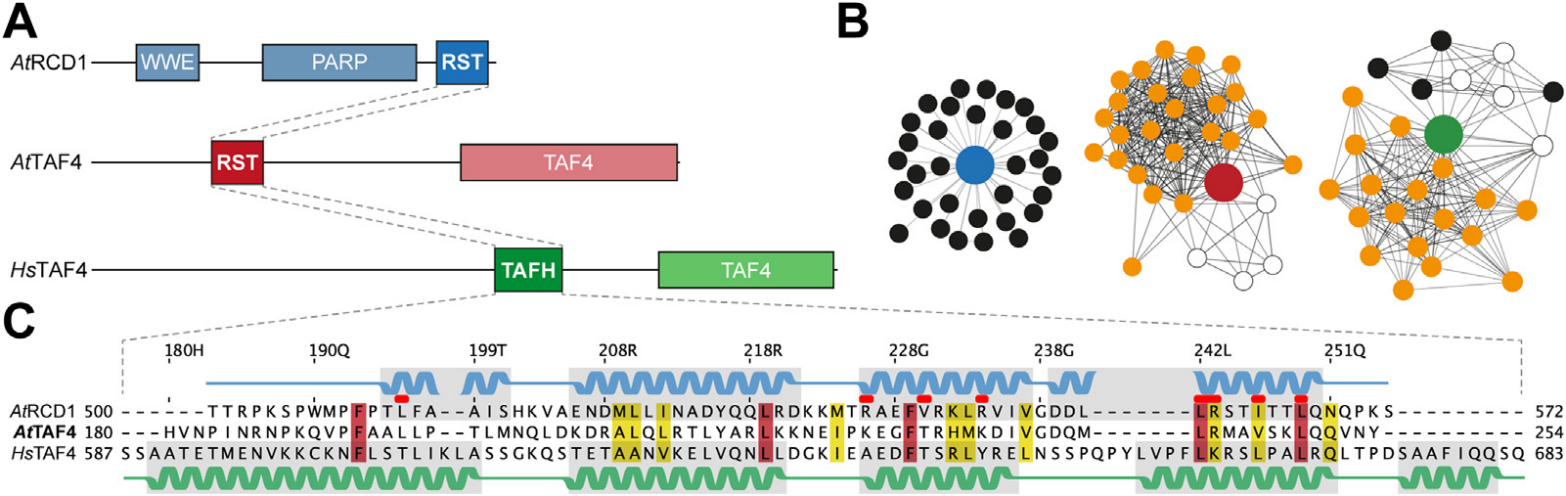
**Domain architectures, interactomes, and sequence alignments of *At*TAF4, *At*RCD1, and *Hs*TAF4.***A*, schematic domain organization of *At*RCD1 (Q8RY59), *At*TAF4 (AT5G43130), and *Hs*TAF4 (O00268). *B*, interactomes of *At*RCD1, *At*TAF4, and *Hs*TAF4 obtained from the IntAct Molecular Interaction Database (60). The central αα-hub containing proteins are color coded as in *A*. *Black* interaction partners are TFs, TFIID components are *orange*, and other types of proteins are shown as *white circles*. *C*, sequence alignment of the *At*TAF4–RST, *At*RCD1–RST, and *Hs*TAF4–TAFH αα-hub domains. Conserved residues are shown in *red*, and positions with conservative substitutions are shown in *yellow*. The secondary structure elements of *At*RCD1–RST (Protein Data Bank code: 5OAO) and *Hs*TAF4–TAFH (Protein Data Bank code: 2P6V) are shown above and below the alignment, respectively. *Red dots* highlight key residues for interactions between *At*RCD1–RST and DREB2A TFs (14). Residue numbering is from *At*TAF4. *At*RCD1, *Arabidopsis thaliana* radical-induced cell death1; *At*TAF4, *Arabidopsis thaliana* transcription initiation factor TFIID-subunit 4; DREB2A, dehydration-responsive element–binding protein 2A; *Hs*TAF4, *Homo sapiens* transcription initiation factor TFIID-subunit 4; TF, transcription factor.

For comparison of protein domains, it is important to consider them as a part of whole proteins. Figure 1*A* shows the domain architectures of the three αα-hub proteins *At*RCD1, *At*TAF4, and *Hs*TAF4. As expected from similarities in functions, the two TAF4 proteins have similar architectures. They carry a C-terminal TAF4 domain, with the αα-hub domain located in slightly different positions within the two proteins. *At*RCD1 has a different domain architecture with the RST αα-hub domain at the C terminus, and WWE (consisting of tryptophan [W] and glutamate [E] residues) and poly(ADP-ribose) polymerase domains characteristic of the SRO family, N-terminal to this (18). Functional similarities and differences are also evident from the interactomes of the three αα-hub–carrying proteins (Fig. 1*B*). *At*RCD1 binds many different TFs (19, 26, 27), while the known interaction partners of the TAF4 proteins are mainly TFIID components, as part of the TFIID complex (32). Thus, the two TAF4 proteins have similar functions and domain architectures, whereas *At*RCD1 is different, both with respect to domain architecture and interactome.

Alignment of the sequences of the three domains revealed low similarity (Fig. 1*C*), with *At*TAF4–RST displaying 25% and 21% identity to the *At*RCD1 and the *Hs*TAF4 αα-hub domains, respectively. Previous studies identified residues involved in *At*RCD1–RST interactions (14, 28). Of these, R560 and I563, which are important for DREB2A binding (14), are conserved or has conservative substitutions in both TAF4 αα-hub domains. For R551, also affecting ligand binding (14), charge conservation in *A*tTAF4–RST (K234), but not in *Hs*TAF4–TAFH (Y641), was seen. According to the structure model of the *At*RCD1–RST–DREB2A complex, V547, L559, and L566 contribute to the hydrophobic ligand-binding cleft of the *At*RCD1–RST αα-hub domain (14). Of these, only the position corresponding to V547 is not conserved in the two TAF4 αα-hub domains, which instead have a threonine in this position (Fig. 1*C*).

In conclusion, *At*TAF4 has a domain architecture and interactome more similar to *Hs*TAF4 than to *At*RCD1. However, sequence comparison revealed residues conserved specifically in the RST domains, suggesting a larger degree of structure–function linkage for these domains. These similarities and differences between the three domains may determine differences in their ligand specificity.

### The *At*TAF4–RST structure reveals a topology similar to *At*RCD1–RST and different from *Hs*TAF4–TAFH

The three-dimensional structures of the *At*RCD1 and *Hs*TAF4 αα-hub domains are known (14, 24, 35). To obtain a structural description of *At*TAF4–RST, we first recorded small-angle X-ray scattering (SAXS) data at six different concentrations (Tables S1 and S2). The shapes of the SAXS curves (Fig. S1*A*) and the Guinier plots (Fig. S1*B*) were similar, indicating the absence of aggregation. *R*_g_ (radius of gyration) and molecular weight (MW) were calculated from the Guinier plots, disregarding data from the lowest and highest concentration (Table S2). An average MW of 8.8 ± 0.4 kDa was obtained, in agreement with the expected MW of 8.9 kDa. The average *R*_g_ was 15.5 ± 0.3 Å, suggesting a slightly more compact structure than that of 16.1 ± 0.2 Å measured for the *At*RCD1–RST domain (14). In addition, the pair distance distribution yielded an average maximal internal distance (*D*_max_) of 44.4 ± 0.7 Å and described a typical globular protein with a short disordered tail, as evidenced by the Gaussian distribution with an asymmetric end (Fig. S1*C*). Finally, the Kratky plots showed a bell shape with a clear maximum indicating a globular fold (Fig. S1*D*).

**Figure 2:**
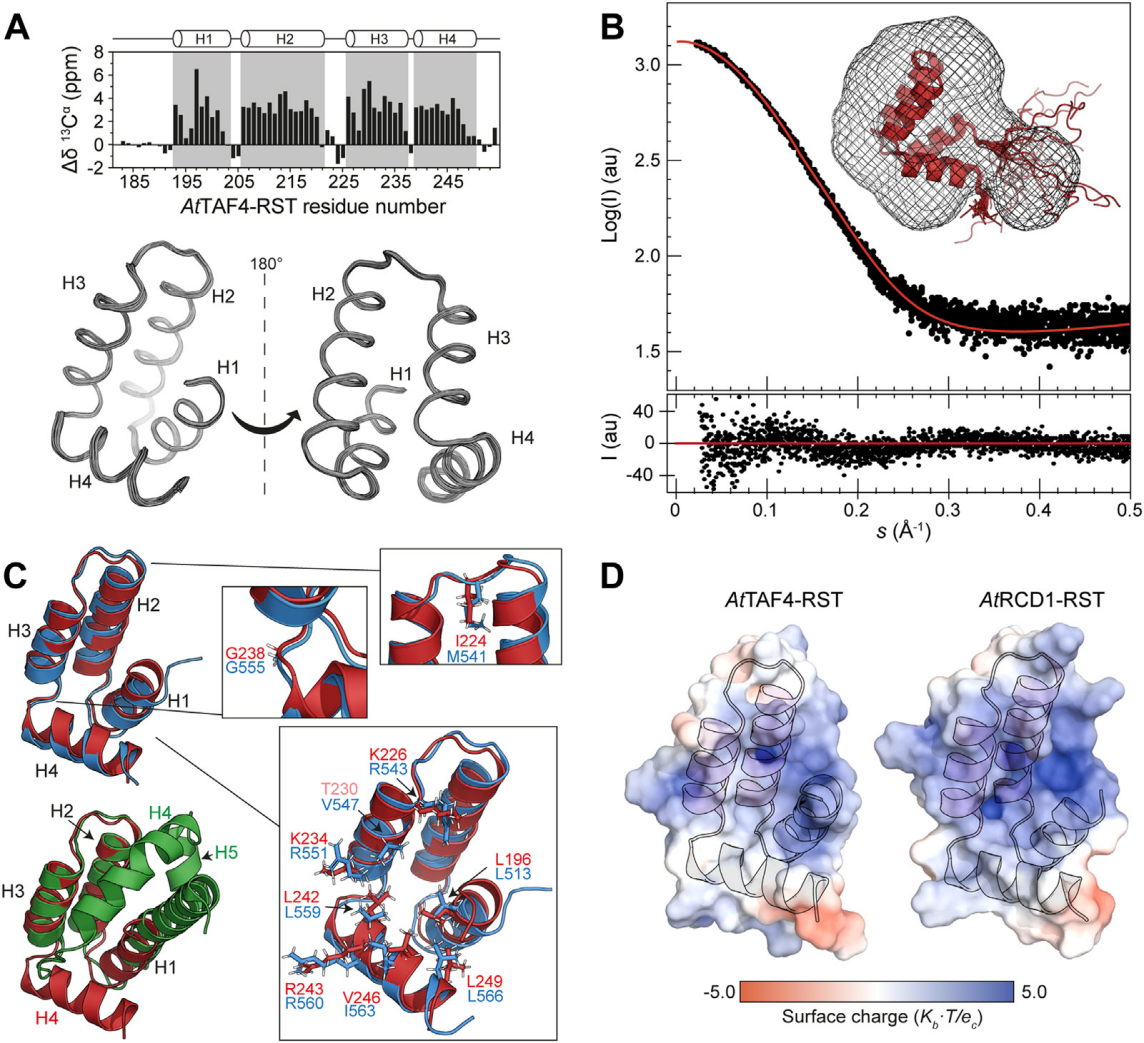
**Structure and SAXS analysis of *At*TAF4–RST.***A*, *top*, secondary C^α^ chemical shifts per residue for *At*TAF4–RST. *Top* schematic shows helix boundaries. *Bottom*, 20 lowest energy structures of *At*TAF4–RST aligned by C^α^ atoms of well-defined region (residues 193–250). *B*, fit of the experimental SAXS curve (4.2 mg/l) (*black*) on the back-calculated SAXS curve obtained from the NMR ensemble (*red line*) using CRYSOL. *Inset*, docking of the NMR structure of *At*TAF4–RST in the *ab initio* averaged bead model envelope. *C*, structure alignments of *At*TAF4–RST (*red*) with *At*RCD1–RST (*blue*) and *Hs*TAF4–TAFH (*green*). *Insets*, residues of *At*TAF4–RST (*red*) and the corresponding residues in *At*RCD1–RST (*blue*) of importance for DREB2A interaction (14), for forming the β3-position, and the tight angle between H3 and H4 is shown as *sticks*. *D*, surface electrostatics of *At*TAF4–RST and *At*RCD1–RST calculated using PyMOL APBS (74). *At*TAF4, *Arabidopsis thaliana* transcription initiation factor TFIID-subunit 4; DREB2A, dehydration-responsive element–binding protein 2A; RST, RCD1, SRO, and TAF4; SAXS, small-angle X-ray scattering; TAFH, TATA-box–associated factor homology.

The structure of *At*TAF4–RST was solved using solution-NMR spectroscopy. A total of 1364 NMR-derived restraints, including 1248 unique distance restraints and 116 dihedral angle restraints (Table S3), were used for calculating a final set of 200 refined structures. Of these, the 20 lowest energy structures without significant violations were selected to represent the structure of the domain (Fig. 2*A*). The SAXS curve obtained from the 4.2 mg/l sample was fitted to a back-calculated scattering curve generated from the NMR ensemble using CRYSOL (part of the ATSAS package (63)) (Fig. 2*B*). The predicted SAXS curve fitted the experimental data well (χ^2^ = 1.19). The comparison between the envelope and the NMR structures, with a χ^2^ = 1.09, confirmed the globular fold of the *At*TAF4–RST domain with the addition of a short disordered tail, here originating from the N-terminal end (Fig. 2*B*).

*At*TAF4–RST consists of four α-helices (H1 [F193–Q203], H2 [K206–K221], H3 [K226–V235], and H4 [D239–Q250]), as described by the secondary ^13^C^α^ chemical shifts (Fig. 2*A*), typical of αα-hub domains (13, 14). The loop connecting H1–H2 consists of two residues, and one residue connects H3–H4. H2 and H3 form the characteristic αα-hairpin supersecondary structure motif consisting of two consecutive antiparallel α-helices connected by a loop (L2). As for prototypical αα-hubs, L2 is folded into the five-residue link motif α_L_–β_4_ (36), with the β3 position of *At*TAF4–RST carrying an isoleucine (I224), as the large hydrophobic side chain interacting with side chains of the two helices (Fig. 2*C*). The four α-helices are organized in the characteristic L-glove (14), in which the hydrophobic surface is exposed to the solvent in an L-shape suitable for protein–protein interactions, similar to that observed in *At*RCD1–RST (Fig. 2*D*).

Structural alignments of *At*TAF4–RST (Protein Data Bank code: 7AC1) with *At*RCD1–RST (Protein Data Bank code: 5OAO) (Fig. 2*C*; C^α^-RMSD = 1.1 Å [54 residues]) revealed almost identical topology and secondary structure, with 15 residues in H2, 12 in H3, and 12 in H4 for both domains, and 11 and 9 residues in H1 of *At*TAF4–RST and *At*RCD1–RST, respectively. The helices of *Hs*TAF4–TAFH are longer, in particular H1, which consists of 25 residues, whereas H2, H3, and H4 consist of 17, 13, and 16 residues, respectively. In addition, *Hs*TAF4–TAFH contains a fifth helix, H5, consisting of nine residues. The L2s are similar in lengths and positions, although the two TAF4 domains have an isoleucine in the αα-hairpin stabilizing β3 position, whereas *At*RCD1–RST has a methionine. As highlighted in the sequence alignment (Fig. 1*C*), the positive charge of the two residues of *At*RCD1–RST participating in electrostatic interactions with *At*DREB2A, R560, and R551, is conserved in *At*TAF4–RST as R243 (H4) and K234 (H3), respectively (Fig. 2*C*). Of the residues engaging in hydrophobic contacts with *At*DREB2A (L513, V547, L559, I563, and L566 of *At*RCD1–RST) (14), L196 (H1), L242 (H4), and L249 (H4), are conserved in *At*TAF4–RST, also with respect to positions in the three-dimensional structure, whereas V547 and I563 are replaced with T230 (H3) and V246 (H4), respectively (Figs. 1*C* and 2*C*). The majority of these are located in H3 and H4. According to the *At*RCD1–RST–DREB2A complex model, mainly H4 is responsible for the interaction with the ligand (14). H4 has the same orientation in *At*RCD1–RST and *At*TAF4–RST (Fig. 2*C*), suggestive of similar ligand-binding clefts. G555 was suggested to be responsible for the tight angle between H3 and H4 of *At*RCD1–RST (28). This position and adjacent residues are conserved in *At*TAF4–RST, whereas it has been replaced by a 6-residue loop in *Hs*TAF4–TAFH. Likely as a result of this difference, H4 has a different orientation in *Hs*TAF4–TAFH compared with the RST domains (Fig. 2*C*). Consequently, the ligand-binding cleft of *Hs*TAF4–TAFH is different from that of the other two αα-hub domains and is located between H1 and H4 (13, 35, 37).

To further compare the structurally similar RST domains, we addressed if the dynamics of the *At*TAF4–RST backbone would also align with that of *At*RCD1–RST (14, 38). This was done by analyzing the longitudinal (*R*_1_) and transverse (*R*_2_) ^15^N relaxation rates and the ^1^H-^15^N HetNOEs (Fig. S2). HetNOEs, reporting on N–H bond dynamics, confirmed the folded core and dynamic flanking regions of *At*TAF4–RST. *R*_1_ relaxation rates were comparable across the chain (*R*_1_ = 1.6 ± 0.1 s^−1^), with a similar pattern displayed by the *R*_2_ relaxation rates (*R*_2_ = 8.7 ± 1.1 s^−1^) (Fig. S2). Elevated *R*_2_ rates were observed for some residues, in particular in H1 and loop regions, indicative of chemical exchange on the millisecond timescale. Compared with the relaxation rate profiles of *At*RCD1–RST (Fig. S2) (39), while mostly similar, marginally higher *R*_1_ rates coupled with generally lower *R*_2_ rates of *At*TAF4–RST suggested a faster global tumbling rate of the *At*TAF4–RST domain, possibly caused by a slightly more compact structure. This is in accordance with SAXS-derived *R*_g_s and structural alignments (Fig. 2*C*). Elevated *R*_2_s may also indicate the presence of a chemical exchange component, as previously shown in *At*RCD1–RST to involve access to an unfolded excited state (38). Analysis of the relaxation rate products (*R*_1_*R*_2_) (Fig. S2*D*), used to decouple global tumbling effects (40), corroborated this. Both RST domains have elevated *R*_2_^15^N relaxation rates of H1 residues, although only *At*RCD1–RST featured the large H1 *R*_2_ value for I517, in the structure positioned opposite to V554/V237, in H3/L3, which in both RST domains have large *R*_2_ values (Fig. S2*E*). No relaxation data are available for *Hs*TAF4–TAFH.

In conclusion, the structure of *At*TAF4–RST revealed an overall topology similar to that of *At*RCD1–RST with corresponding secondary structure, helix orientations, and putative ligand-binding cleft. This is in contrast to the topology of *Hs*TAF4–TAFH, which has a fifth helix and a different H4 orientation, all suggestive of a different ligand-binding site. The two RST domains have overall similar dynamical behavior, but global differences indicate a faster global tumbling rate of the *At*TAF4–RST domain, originating from a more compact overall structure.

### The three αα-hub domains have a common thermal unfolding state but different thermodynamic features

Conformational stability and interactome size have been hypothesized to correlate in a way that more stable proteins infer smaller interactomes (41, 42). Thus, considering the differences in dynamics highlighted in the previous paragraph, we determined the conformational stability of the three domains. Their conformational stabilities were determined in chemical and thermal denaturation experiments, and the unfolding process followed by CD spectroscopy, *via* the change in ellipticity at 222 nm, and by two-dimensional global analysis of the change in intrinsic fluorescence (Fig. S3). While the CD experiments monitor the change in secondary structure in response to increasing temperature or urea concentration, the intrinsic fluorescence follows the chemical environment of the aromatic residues (43), typically reflecting the tertiary structures. The latter was analyzed by a two-dimensional fitting procedure that combines temperature and chemical denaturant unfolding (44). Both denaturation processes produced a sigmoidal curve characteristic of a two-state unfolding (Fig. S3). Table 1 shows the parameters determined from the experiments.

**Table 1:** Stability of αα-hub domains

Domain/method	*T*_m_ (°C)^a^	*ΔH*^b^ (kJ mol^−1^)	*ΔC*_p_ (kJ mol^−1^ K^−1^)	*m* (kJ mol^−1^ M^−1^)	*ΔG*_DN_^c^ (kJ mol^−1^)
*At*TAF4–RST					
CD chemical denaturation				3.7 ± 0.7	7 ± 2
CD thermal denaturation	68 ± 3	46 ± 6			
Two-dimensional global analysis	66 ± 2	117 ± 26	3.1 ± 0.6	3.4 ± 0.3	5.9 ± 1.3
*At*RCD1–RST					
CD chemical denaturation				2.7 ± 0.3	7.0 ± 0.6
CD thermal denaturation	68.8 ± 0.2	54.8 ± 0.9			
Two-dimensional global analysis	59 ± 5	118 ± 13	1.8 ± 0.3	3.8 ± 0.2	8.8 ± 1.4
*Hs*TAF4–TAFH					
CD chemical denaturation				5.5 ± 0.2	13.2 ± 0.4
CD thermal denaturation	74.1 ± 0.4	55 ± 2			
Two-dimensional global analysis	71 ± 3	167 ± 16	3.4 ± 0.1	4.1 ± 0.1	12.9 ± 1.4

Chemical denaturation was performed by increasing the concentration of denaturant from 0 to 8 M urea, whereas thermal denaturation was performed by increasing the temperature from 15 to 90 °C. The thermodynamic parameters were calculated using Equations 2 (CD) and 3 (two-dimensional global analysis). The values are averages and standard deviations of three independent experiments.

a In the absence of denaturant.

b *ΔH* corresponds to the *ΔH*_vH_ for the CD thermal denaturation and *ΔH*_m_ for two-dimensional global analysis.

c Value calculated at 25 °C.

**Figure 3:**
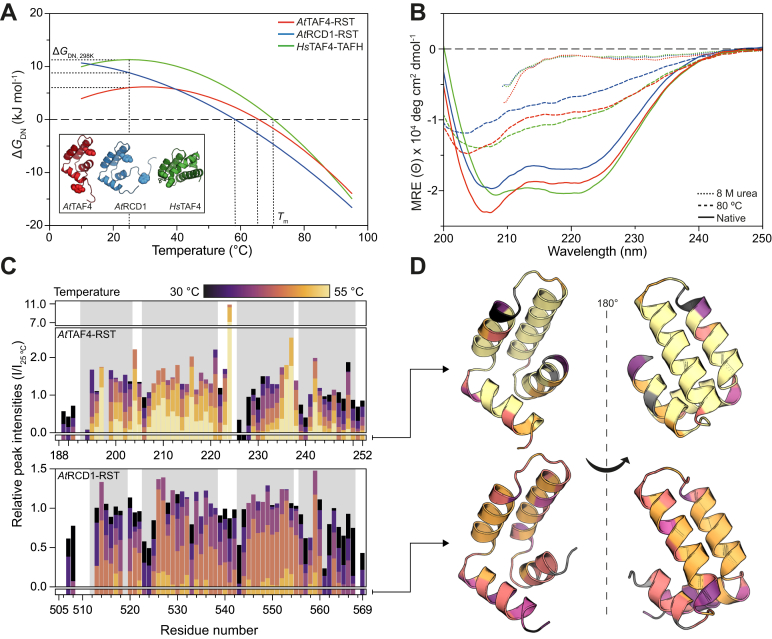
**Stability of αα-hub domains.***A*, stability curves of *At*TAF4–RST (*red*), *At*RCD1–RST (*blue*), and *Hs*TAF4–TAFH (*green*) calculated from two-dimensional global analysis according to Equation 6. *Inset*, position of intrinsic fluorophores (tyrosine and tryptophan) used to monitor unfolding. *B*, CD spectra of 1 mg ml^−1^*At*RCD1–RST (*blue*), *At*TAF4–RST (*red*), and *Hs*TAF4–TAFH (*green*) acquired in 20 mM sodium phosphate, pH 7.4, at 20 °C (*solid line*), 80 °C (*dashed line*), and in buffer containing 8 M urea (*dotted line*) at 20 °C. The data recorded in the presence of urea were excluded below 205 nm because of HT >600 V. *C*, relative peak intensities in ^15^N,H HSQC spectra of *At*TAF4–RST (*top*) and *At*RCD1–RST (*bottom*) at increasing temperatures from 30 to 55 °C, with the intensity at 25 °C as reference. The *bars* below the column graphs indicate the highest temperature for which a peak could be identified. *D*, residues colored according to highest temperature for which a peak could be assigned (from *C*) mapped to the structure of *At*TAF4–RST (*top*) and *At*RCD1–RST (*bottom*). *Gray colors* are unassigned residues. *At*RCD1, *Arabidopsis thaliana* radical-induced cell death1; *At*TAF4, *Arabidopsis thaliana* transcription initiation factor TFIID-subunit 4; HSQC, heteronuclear single quantum coherence; *Hs*TAF4, *Homo sapiens* transcription initiation factor TFIID-subunit 4; RST, RCD1, SRO, and TAF4; TAFH, TATA-box–associated factor homology.

According to the two-dimensional global analyses of the unfolding reaction followed by fluorescence (Fig. 3*A*), the *At*TAF4–RST domain had a *T*_m_ of 66 ± 2 °C and a free energy of unfolding (*ΔG*_DN, 298K_) of 5.9 ± 1.3 kJ mol^−1^, suggestive of a low stability. The parameters determined by CD spectroscopy were *T*_m_ = 68 ± 3 °C and *ΔG*_DN, 298K_ = 7 ± 2 kJ mol^−1^. The *m* values, proportional to the change in solvent-accessible surface area upon unfolding (45), were 3.7 ± 0.7 kJ mol^−1^ M^−1^ and 3.4 ± 0.3 kJ mol^−1^ M^−1^, determined by CD and fluorescence spectroscopy, respectively. These values correspond to the unfolding of approximately 45 residues and exposure of 3300 Å^2^ surface (45). A small unfolding heat capacity change (Δ*C*_p_) of 3.1 ± 0.6 kJ mol^−1^ K^−1^ is in accordance with the high *T*_*m*_ despite the low unfolding energy, since it causes a decrease in the temperature dependence of the stability (Fig. 3*A*).

For comparison, we determined the parameters for unfolding of the *Hs*TAF4–TAFH and *At*RCD1–RST domains (Table 1 and Fig. S3). Figure 3*A* shows the three stability curves obtained from the two-dimensional global analyses. The global stability, Δ*G*_DN_ of the two αα-hub domains from the TAF4 proteins, had similar temperature dependence, described by the unfolding Δ*C*_p_s, but with *Hs*TAF4–TAFH having a higher enthalpy change, Δ*H*_m_, in accordance with the increased stability of *Hs*TAF4–TAFH compared with that of *At*TAF4–RST. *At*RCD1–RST had a Δ*H*_m_ similar to that of *At*TAF4–RST, but a lower Δ*C*_p_ of 1.8 ± 0.3 kJ mol^−1^ K^−1^, reflecting the lower temperature dependence of Δ*G*_DN_. The Δ*H*_m_ was larger for *Hs*TAF4–TAFH than for both RST domains because of more folded residues in this larger domain. This difference also resulted in a higher *T*_m_ of 71 ± 3 °C for *Hs*TAF4–TAFH compared with 66 ± 2 °C for *At*TAF4–RST and 59 ± 5 °C for *At*RCD1–RST (Fig. 3*A*).

From the CD experiments, *Hs*TAF4–TAFH also had a higher *T*_m_ = 74.1 ± 0.4 °C, compared with *T*_m_ = 68.8 ± 0.2 °C for *At*RCD1–RST and *T*_m_ = 68 ± 3 °C for *At*TAF4–RST, respectively. A similar order was observed when comparing Δ*G*_DN, 298K_ values; those of the RST domains were similar, whereas the TAFH domain had significantly larger Δ*G*_DN, 298K_. The smallest *m* values were obtained for *At*RCD1–RST and *At*TAF4–RST, being 2.7 ± 0.3 kJ mol^−1^ M^−1^ and 3.8 ± 0.7 kJ mol^−1^ M^−1^, respectively, with a higher *m* value of 5.5 ± 0.2 kJ mol^−1^ M^−1^ for *Hs*TAF4–TAFH, indicating a larger change in solvent-accessible surface area upon unfolding (Table 1). Although the two types of experiments do not measure the same features, there is accordance between the results obtained (Table S4).

The analyses showed that the *ΔH*s extracted from the two different unfolding experiments were different. For all three domains, the van’t Hoff enthalpy change, *ΔH*_vH_, determined from the CD thermal denaturation was lower than the enthalpy change determined using the two-dimensional global analyses, Δ*H*_m_ (ratios ∼0.5, Tables 1 and S4), suggesting incomplete thermal unfolding and thus the presence of a putative alternative state for all three αα-hub domains. To explore this further, and to obtain structural information on this alternative state, we obtained CD spectra before and after chemical and thermal denaturation and observed the existence of pronounced residual helical structure at high temperature. In contrast, the domains were completely denatured in 8 M urea (Fig. 3*B*). We calculated the percentage helicity of the three domains at 20 °C and compared with those calculated for the denatured states. At 20 °C, the domains were ∼50% helical (43% for *At*RCD1–RST, 49% for *At*TAF4–RST, and 52% for *Hs*TAF4–TAFH). In the presence of 8 M urea, all domains were extensively unfolded with only ∼3% helicity. However, at 80 °C in the thermally denatured states, the domains retained ellipticity at 222 nm corresponding to 17%, 23%, and 25% helical structure for *At*RCD1–RST, *At*TAF4–RST, and *Hs*TAF4–TAFH, respectively. Previous observations indicate that thermally and chemically denatured protein may differ because of subensembles populated at high temperatures (46, 47). However, for the TAF4 αα-hub domains, a comparably more negative ellipticity than observed for thermal denaturation in general (47) indicates retention of some α-helical structure in the unfolded state.

To further address the characteristics of the alternative state, and since hydrogen-exchange kinetics of the *At*RCD1–RST domain has been shown to be extremely fast (38), we recorded series of ^15^N-heteronuclear single quantum coherence (HSQC) spectra of *At*TAF4–RST and *At*RCD1–RST over a temperature range from 25 to 55 °C (Fig. S4). For *At*TAF4–RST, peak intensities increased considerably up until 40 to 45 °C (Fig. 3*C*). Although surprising, this matched the relative temperature independence of Δ*G*_DN_ in this temperature range (Fig. 3*A*), resulting in limited unfolding but faster global tumbling, which produced sharper peaks in the HSQC spectra. At 45 to 55 °C, most peaks lost intensity although the majority of *At*TAF4–RST peaks could be assigned even at the highest temperature. The behavior of *At*RCD1–RST was distinctly different with most residues losing peak intensity as temperature increased above 30 °C, and no peaks were visible at temperatures above 50 °C (Fig. 3*C*). Interestingly, peaks belonging to residues in H2 and H3, constituting the αα-hairpin, were more temperature resistant than peaks from the flanking helices. This suggested that the hydrogen bonds of the H2 and H3 were retained at higher temperatures, thus limiting solvent proton exchange of the backbone amide groups. A similar pattern was not immediately apparent for *At*TAF4–RST. However, mapping of the highest temperature for which a peak from a particular residue was visible (Fig. 3*D*) revealed that peaks from solvent-exposed residues, particularly of H1 and H4, generally disappeared at lower temperature, whereas peaks from residues facing the “interior” of the protein were visible at higher temperatures. This effect was also seen for *At*RCD1–RST, although to a lesser extent.

Based on the results presented previously, three conclusions can be made. First, *Hs*TAF4–TAFH is the most stable domain, most likely because of a larger buried surface area and a higher number of folded residues. Second, the unfolding *ΔC*_p_ of *At*RCD1–RST is smaller than that of *At*TAF4–RST even though they are structurally and sizewise similar. Finally, the discrepancies between *ΔH*_m_ and Δ*H*_vH_ values, together with the presence of residual structure after thermal unfolding, suggested the existence of a general αα-hub thermal unfolding state. This supports the presence of stable cores, inferred from the NMR data to be comprised primarily of H2 and to some extent H3, organizing more dynamic flanking helices.

### *At*TAF4–RST interacts with the *At*RCD1 ligands *At*DREB2A and ANAC013

**Figure 4:**
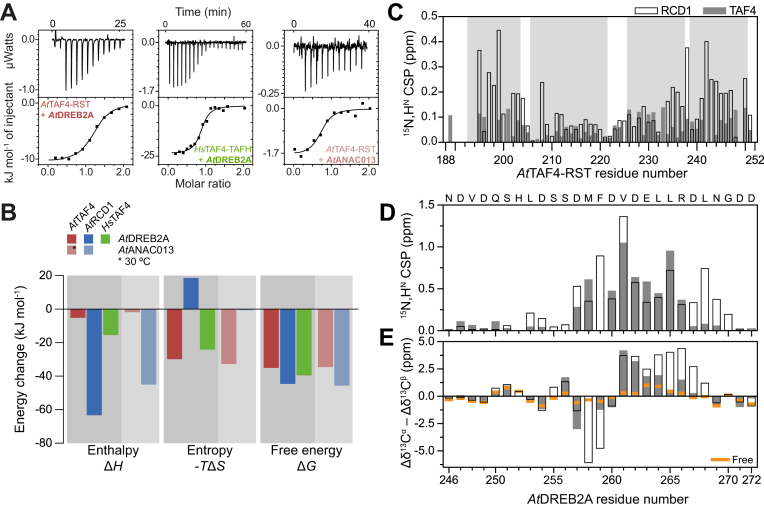
**Transcription factor binding to αα-hub domains.***A*, ITC data showing the titration of *At*TAF4–RST into DREB2A_243–272_ (*left*), *Hs*TAF4–TAFH into DREB2A_243–272_ (*middle*), and *At*TAF4–RST into ANAC013 (*right*). Experiments were performed at 30 °C. For each experiment, the *upper panel* shows baseline-corrected raw data from the titration, and the *lower panel* shows the integrated peaks and the fitted binding curve. *B*, thermodynamic parameters of the interaction of three αα-hubs with *At*DREB2A (*dark*) and *At*ANAC013 (*light*) derived from ITC experiments shown in *A* and for Fig. S5 and from experiments shown in Refs. (27, 28). *C*, *At*TAF4–RST ^15^N,H^N^ CSPs (*gray bars*) induced upon binding of *At*DREB2A_243–272_ shown along with *At*RCD1–RST (*hollow bars*, data from Ref. (39)) using TAF4–RST residue numbering. *D*, *At*DREB2A ^15^N,H^N^ CSPs for binding *At*TAF4–RST (*gray bars*) (Fig. S6) and *At*RCD1–RST (*hollow bars*, data from Ref. (39)). The sequence of *At*DREB2A is shown at the *top*. F259 could not be assigned in the bound state of *At*TAF4–RST. *E*, ^13^C secondary chemical shifts in the free state (*orange*) and in *At*TAF4–RST (*gray bars*) and *At*RCD1–RST (*hollow bars*) bound states. For the *At*TAF4–RST, M258 could not be assigned, and only ^13^C^α^ was visible for F259. ANAC, *A. thaliana* NAM, ATAF1/2, and CUC2; *At*TAF4, *Arabidopsis thaliana* transcription initiation factor TFIID-subunit 4; CSP, chemical shift perturbation; DREB2A, dehydration-responsive element–binding protein 2A; *Hs*TAF4, *Homo sapiens* transcription initiation factor TFIID-subunit 4; ITC, isothermal titration calorimetry; RST, RCD1, SRO, and TAF4; TAFH, TATA-box–associated factor homology.

*At*DREB2A has previously been identified as an *At*RCD1–RST interaction partner (19, 26, 27, 48). Based on the similarities of the structures and conservation of key ligand-interacting residues, we hypothesized that *A*tTAF4–RST would also bind *At*DREB2A. Using isothermal titration calorimetry (ITC), the interaction between an *At*DREB2A peptide DREB2A_243–272_, containing the RCD1-binding SLiM (27) and *At*TAF4–RST, was analyzed. We performed two sets of experiments; one at 25 °C (Fig. S5), which suggested an interaction between *At*TAF4–RST and DREB2A_243–272_, but with a small change in binding enthalpy, and another at 30 °C, which confirmed binding and had an increased contribution from Δ*H* and thus an improved signal-to-noise ratio (Fig. 4*A*). The *K*_*d*_s for the complex of DREB2A_243–272_ with *At*TAF4–RST were at 25 and 30 °C 740 ± 300 nM and 1050 ± 140 nM, respectively. The interactions were characterized by a low enthalpic contribution (Δ*H* = −5.19 ± 0.03 kJ mol^−1^ at 25 °C and −10.5 ± 0.2 kJ mol^−1^ at 30 °C) and were in both cases dominantly driven by entropy (−*T*Δ*S* = −29.8 kJ mol^−1^ at 25 °C and −24.2 kJ mol^−1^ at 30 °C) (Table 2 and Fig. 4*B*). The significant difference in entropy between the *At*TAF4–RST and *At*RCD1–RST interactions with *At*DREB2A suggested that the *At*TAF4 interaction involved less structuring than the *At*RCD1 interaction.

**Table 2:** Thermodynamic analysis of interactions

Syringe/cell	Temperature (°C)	*K*_*d*_ (nM)	N	Δ*H* (kJ mol^−1^)	−TΔ*S* (kJ mol^−1^)	Δ*G* (kJ mol^−1^)
*At*TAF4–RST/DREB2A	25	740 ± 320	1.24 ± 0.04	−5.19 ± 0.03	−29.8	−35.0
*At*TAF4–RST/DREB2A	30	1050 ± 140	1.18 ± 0.01	−10.5 ± 0.2	−24.2	−34.7
*At*RCD1–RST/DREB2A^a^	25	16 ± 1	0.90 ± 0.00	−63.3 ± 0.2	18.7	−44.6
*Hs*TAF4–TAFH/DREB2A	25	110 ± 50	1.15 ± 0.02	−15.4 ± 0.4	−24.1	−39.5
*Hs*TAF4–TAFH/DREB2A	30	420 ± 120	0.85 ± 0.02	−23.9 ± 0.7	−12.9	–36.9
*At*TAF4–RST/ANAC013	25	NB	NB	NB	NB	NB
*At*TAF4–RST/ANAC013	30	1080 ± 630	0.68 ± 0.04	−1.8 ± 0.1	−32.7	−34.5
*At*RCD1–RST/ANAC013^b^	25	9 ± 4	0.80 ± 0.01	−45.0 ± 0.8	−0.6	−45.6

Abbreviation: NB, no detectable binding.

Syringe/cell indicates whether the αα-hub domain or the TF is the titrant in the syringe or the titrant in the cell. The standard errors for Δ*H*, *K*_*d*_, and N were obtained from Origin when fitting the data to a model of one set of binding sites.

a Data from Ref. (28).

b Data from Ref. (27).

To characterize the interaction between *At*TAF4–RST and DREB2A_243–272_ at the residue level, we used NMR spectroscopy (Fig. 4*C*). *At*TAF4–RST was in fast-intermediate exchange between free and bound states on the NMR timescale enabling assignment of the bound state. Most *At*TAF4–RST residues were affected by binding, suggesting binding to be accompanied by small structural rearrangements or stabilization of the *At*TAF4–RST α-helices, as seen previously for *At*RCD1–RST (14). For *At*RCD1–RST, most key residues for binding of *At*DREB2A map to H3 and H4 (13, 14). Comparison of free and bound states of *At*TAF4–RST revealed larger chemical shift perturbations (CSPs) in H3 and H4, suggesting that for *At*TAF4–RST, these regions are also involved in binding, further supporting the ligand-binding cleft shown in Figure 2*D*. However, the CSPs were generally smaller for the *At*TAF4–RST interaction than for the interaction of *At*RCD1–RST, suggesting that *At*TAF4–RST undergoes reduced structural changes upon *At*DREB2A binding.

NMR spectroscopy was used to study the structural features of the *At*TAF4–RST–bound state of DREB2A_243–272_ (Fig. S6). Residues from D257 to R266 showed large CSPs upon binding (Fig. 4*D*), similar to the results described for the *At*RCD1–RST–DREB2A complex (27). Secondary ^13^C chemical shifts indicated helical structure in the bound-state DREB2A peptide (Fig. 4*E*). However, the *At*TAF4–RST binding-induced α-helix was shorter than when in complex with *At*RCD1–RST (14, 39). In addition, the secondary chemical shift of the highly conserved F259 (28), which forms extended structure in complex with *At*RCD1–RST (39), did not exhibit the same behavior in complex with *At*TAF4–RST. Peaks belonging to residues M258 and F259 were very weak or nonexistent indicating that the two residues were dynamic in the *At*TAF4–RST complex. This is similar to what was observed for a shorter, lower affinity, DREB2A_255–272_ fragment in complex with *At*RCD1–RST (14, 39). Together, this suggested that for DREB2A_243–272_, binding to *At*TAF4–RST induced helical structure in the 261 to 264 region of the peptide, while the residues surrounding this central helical turn remained unstructured, a clear contrast to the interaction with *At*RCD1–RST.

**Figure 5:**
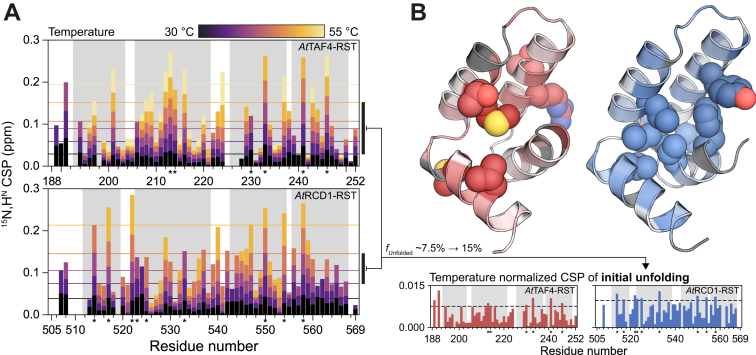
**Initial unfolding of RST domains based on NMR temperature denaturation.***A*, ^15^N,H^N^ chemical shift perturbations (CSPs) for *At*TAF4–RST (*top*) and *At*RCD1–RST (*bottom*) as a function of increasing temperature. *Colored horizontal lines* indicate the upper quartile CSP of the temperature. The *vertical black bars* on the *right* indicate the temperature range used to probe initial unfolding CSPs. *B*, temperature normalized CSPs of a temperature range corresponding to a change from ∼7.5% to ∼15% unfolded for *At*TAF4–RST (*red*) and *At*RCD1–RST (*blue*). A threshold of mean + 1 standard deviation (*dashed line*) was used to highlight residues (*spherical representation*) experiencing larger CSPs than others. For *At*RCD1–RST, these congregate around the interface with helix 1. *At*RCD1, *Arabidopsis thaliana* RCD1; *At*TAF4, *Arabidopsis thaliana* transcription initiation factor TFIID-subunit 4; RST, RCD1, SRO, and TAF4.

Both thermodynamics parameters and the secondary chemical shifts indicated less structuring of DREB2A_243–272_ in the *At*TAF4–RST interaction compared with the interaction with *At*RCD1–RST. To rationalize the different potential for structuring of the two RST domains, we analyzed the NMR unfolding CSPs in context of their respective thermal stabilities (Figs. 3*A*, S4 and Table 1). Based on Δ*G*_DN_ temperature dependence (Equation 6, no denaturant), we determined temperature ranges resulting in similar degrees of unfolding (30–50 °C for *At*TAF4–RST and 35–45 °C for *At*RCD1–RST). If the domains contain structure with lower stability, we expect adjacent residues to experience increased CSPs in the selected temperature range. The extracted CSPs were generally larger for *At*RCD1 than for *At*TAF4. In addition, *At*RCD1–RST showed a clear increase in CSPs for residues located in the interface between H1 and the remaining folded domain (Fig. 5*B*). For *At*TAF4–RST, the CSPs were less localized. Comparison of the initial unfolding of the two domains suggested that H1 of *At*RCD1–RST was more sensitive to temperature and thus also more malleable. This malleability would allow *At*DREB2A to induce specific complementary structure, thus increasing the favorable enthalpic contribution at an entropic cost.

We then examined how the differences in topology and the lack of *At*RCD1–RST key residues (Figs. 1*C* and 2*C*) in *Hs*TAF4–TAFH would affect binding to DREB2A_243–272_ (Figs. 4*B* and S5*B*; Table 2). Surprisingly, the affinity of *Hs*TAF4–TAFH for DREB2A_243–272_ was higher (*K*_*d*_ = 110 ± 50 nM at 25 °C) than that of *At*TAF4–RST (*K*_*d*_ = 740 ± 320 nM) and was driven by both enthalpy and entropy, with the largest contribution stemming from entropy changes. The binding cleft of the TAFH domain differs from that of the two RST domains by being located between H1 and H4, rather than between H3 and H4 (37). Formation of a coactivator–TF complex may thus in this case be explained by stochastic burial of hydrophobic residues and unspecific electrostatic interactions, as commonly assumed for such interaction pairs (49).

Finally, we measured the binding of *At*TAF4–RST to the *At*ANAC013 peptide, ANAC013_254–274_. This peptide also contains the *At*RCD1-binding SLiM but behaves structurally differently from DREB2A_243–272_, with no detectable α-helix induction upon binding to *At*RCD1–RST (27). In this experiment, the *ΔH* measured at 25 °C by ITC was too low for detection (Fig. S5*C*), but the experiment performed at 30 °C (Fig. 4, *A* and *B*) allowed determination of the thermodynamic parameters associated with binding. *At*TAF4–RST bound ANAC013_254–274_ with *K*_*d*_ 1080 ± 630 nM, a small enthalpic contribution (Δ*H* = −1.8 ± 0.1 kJ mol^−1^), and a large favorable contribution from entropy change to binding (−*TΔS* = −32.7 kJ mol^−1^) (Table 2).

To conclude, the interactions between *At*TAF4–RST and two *At*RCD1-binding TFs were mainly driven by favorable changes in entropy, which is in contrast to their *At*RCD1–RST interactions. For the *Hs*TAF4–TAFH–DREB2A_243–272_ interaction, entropy also gave the largest contribution to binding at 25 °C (Table 2). Structural analysis suggested that although DREB2A_243–272_ undergoes coupled folding and binding in its interaction with *At*TAF4–RST, the resulting α-helix is shorter than in the *At*RCD1–RST complex.

## Discussion

In this work, we have asked which properties within interactomes are important for selectivity and specificity. To address this, we have investigated the *At*TAF4–RST αα-hub domain and compared it with two other αα-hub domains, one from the same species and one from humans. *At*RCD1–RST was included because it is also an RST domain (13, 48), but its parent protein, *At*RCD1, belongs to a different functional family than TAF4 (18). *Hs*TAF4–TAFH was therefore also included, as it represents a TAF4 protein, but from a different species (Fig. 1*A*). Evolutionarily, the RST and TAFH domains differ from the paired amphipathic helix, harmonin–homology domain, and nuclear coactivator–binding domain αα-hubs by having an intron just before the region encoding the α_L_–β_4_ motif (13). Despite this, there is no evidence of a common ancestor, and thus the evolutionary relation between the two genes remains uncertain.

As a first step, the three-dimensional structure of *At*TAF4–RST was determined and compared with the structures of the other two αα-hubs. The two RST domains were structurally similar forming an L-glove fold with four helices of similar lengths and the linker between H2 and H3 forming the α_L_–β_4_ motif (Fig. 2*C*). The RST domain structures are different from that of *Hs*TAF4–TAFH with respect to the orientation of H4. Together with the different lengths of H1, the varying H4 orientations represent the distinctive features of different αα-hub subgroups (13, 14). Noteworthy, this changes the binding surface of *Hs*TAF4–TAFH compared with the two RST domains (Fig. 2, *C* and *D*). The *At*RCD1–RST–DREB2A complex is stabilized by residues L513, R543, V547, R551, L559, R560, I563, and L566 of *At*RCD1–RST (14). All these positions, except V547, are conserved or have conservative substitutions in *At*TAF4–RST (Figs. 1*C* and 2*C*). Thus, the lack of specific charged and hydrophobic residues does not explain the difference in affinities between the two RST domains, suggesting that specificity is acquired from differences in other properties of the two domains.

**Figure 6:**
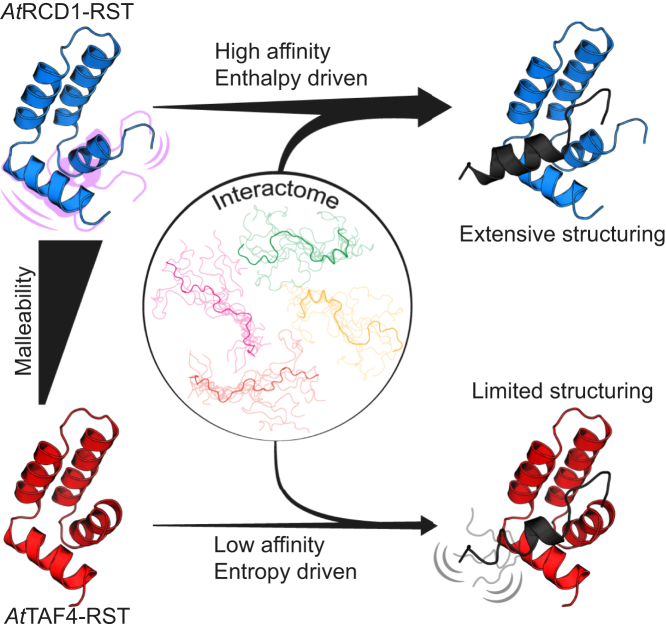
**Model for αα-hub–TF interactions.** The degree of structural dynamics and flexibility in the hub may be deterministic for binding of multiple ligands with high affinity through cooperative coupled folding and binding. This is illustrated using the interactions of two different RST domains with the disordered TF *At*DREB2A. The malleable *At*RCD1–RST domain binds *At*DREB2A with high affinity resulting in considerable structuring of both proteins, whereas the less dynamic *At*TAF4–RST binds *At*DREB2A with lower affinity and much less structuring of DREB2A. *At*DREB2A, *Arabidopsis thaliana* dehydration-responsive element–binding protein 2A; *At*RCD1, *Arabidopsis thaliana* RCD1; *At*TAF4, *Arabidopsis thaliana* transcription initiation factor TFIID-subunit 4; RST, RCD1, SRO, and TAF4; TF, transcription factor.

The conformational stability of protein hubs has recently been hypothesized to be important for their functions as exemplified by a correlation between malleability and promiscuity (49, 50). Analyzing the stability and folding thermodynamics of the three αα-hubs, we found that the domains populate a common alternative state at high temperatures with relatively high content of helicity (Fig. 3*C* and Table 1). It is possible that this state represents the unfolded excited state, recently identified for *At*RCD1–RST (38), but more elaborate studies are needed to fully confirm this. Previous studies on the dynamics and stability of αα-hubs suggested that the H2–H3 αα-hairpin comprises a relatively rigid structural element, whereas the other helices, in particular the C-terminal region of H1, are more flexible (13). Thermal denaturation using CD and NMR spectroscopy corroborated this (Fig. 3, *B*–*D*). Although similarities concerning the unfolding state can be identified, our data indicate that the two RST domains behave differently when exposed to higher temperatures, with *At*TAF4–RST being relatively rigid and maintaining its structure, whereas *At*RCD1–RST has more flexible flanking helices that unfold at lower temperatures. We suggest that these features provide the foundation of the larger interactome of *At*RCD1–RST as it allows the hub protein to adapt and bind an increased number of interaction partners with higher affinity (Fig. 6).

Heat capacity depends on many parameters including hydration of hydrophobic groups, electrostatics, hydrogen bonding, and conformational entropy (51). The two RST domains have similar folds and conformational stabilities but exhibit different unfolding Δ*C*_p_s. Since the *At*RCD1–RST domain is more dynamic and malleable in the native state, it is possible that the core of *At*RCD1–RST exposes more hydrophobic surface than *At*TAF4–RST in the native state, resulting in the smaller Δ*C*_p_ upon denaturation. The increased malleability of the native state, together with larger temperature dependence of stability (Fig. 3*A*), would ensure functionality in a large range of environments and with a large number of different TF ligands, as in the RCD1–interactome (13). In contrast, based on the observation that the *At*TAF4–RST stability was less temperature dependent (Fig. 3*A*) and that the CSPs of initial unfolding were smaller and more widely distributed (Fig. 5), it is possible that *At*TAF4–RST confers more narrow specificity (Fig. 1*B*) associated with more specific functional roles (Fig. 6). *Hs*TAF4–TAFH was the most stable of the three αα-hub domains. This could be due to the larger size (39 residues longer) with more folded residues resulting in a larger Δ*H* and a larger *m* value and a corresponding lower flexibility and could analogously explain its fewer known interaction partners (Fig. 1*B*).

Specificity for AD–coactivator interactions remains an intriguing question, dominated by the acceptance of functional interchangeability of ADs and coactivators (3, 4, 5), although with recent suggestions of specificity in these types of interactions (10, 12). Here, we analyzed the ability of the αα-hub domains to bind *At*DREB2A. *At*DREB2A is a biological ligand of *At*RCD1, and interactions between *At*RCD1 and *At*DREB2A negatively regulates *At*DREB2A (27, 52). *At*DREB2A bounds *At*RCD1–RST with high affinity but bounds also both *At*TAF4–RST and *Hs*TAF4–TAFH with affinities typical of αα-hub–TF interactions (13) (Table 2). However, whereas the *At*RCD1–RST–DREB2A interaction was driven by enthalpy with a considerable entropic penalty, the interactions of *At*DREB2A with the other αα-hub domains were driven by large favorable entropic contributions. This was especially pronounced for the interaction with *At*TAF4–RST, for which the enthalpic contribution was very low and binding-induced folding reduced compared with the interaction with *At*RCD1–RST (Fig. 6). Speculating, thermodynamics may be a route to distinguish biological ligands from nonspecific ligands. *At*DREB2A, as a biological ligand of *At*RCD1–RST, forms an extensive network of specific noncovalent bonds with *At*RCD1–RST (13, 14), absent in complex with the two other αα-hubs. In these cases, retained flexibility rather than noncovalent bonds may drive the interactions through a reduced loss of conformational entropy (13, 14). In accordance with a general model for intrinsic disorder–based interactions (53), the disordered RCD1-binding SLiM of DREB2A would initially bind all three αα-hub domains in multiple different conformations, likely using interaction hot spot residues (27, 54). Then, only in complex with *At*RCD1–RST, it would fold cooperatively with *At*RCD1–RST into a native complex with extensive formation of specific noncovalent bonds as well as more helix stabilization in the hub itself (Fig. 6). In this case, changes in binding enthalpy govern high-affinity complex formation, potentially leading to longer lifetimes of the biologically relevant complexes. In other cases, entropy may also be important for formation of high-affinity complexes (55, 56, 57). Together, the results show how conformational flexibility of intrinsic disorder contributes to protein–protein interactions by allowing partner adaptation (16, 53) and how the balance between binding enthalpy and entropy may fine-tune affinity but more importantly, specificity of AD–coactivator interactions.

In contrast to *At*DREB2A, ANAC013 does not fold when binding to *At*RCD1–RST (27, 28). The high affinity of ANAC013 for *At*RCD1–RST is still sustained by binding enthalpy (27) (Table 2), whereas the two orders of magnitude weaker complex of ANAC013 with *At*TAF4–RST is based on favorable entropic contributions. This raises the questions of whether ANAC013, also regulated by interactions with *At*RCD1 in plant stress responses (24, 25), is indeed an *in vivo* ligand of *At*TAF4. The expression patterns of the *AtDREB2A* and the *ANAC013* genes are similar and induced in response to various hormones and stressors (20), whereas the *AtRCD1* and *AtTAF4* genes are constitutively expressed (30, 58). Even though induced levels of the TFs may enable low-affinity interactions to take place *in vivo*, *At*RCD1 is likely to outcompete *At*TAF4 for TF interactions. If *At*DREB2A and ANAC013 are not *in vivo* ligands of *At*TAF4–RST, what are then the ligands? Based on functional similarities of *Arabidopsis* and human TAF4, *At*TAF4–RST may also exert narrow selectivity in interactions (13).

In this study, we explored the properties within interactomes that could be relevant for selectivity in hubs. We determined the structure of the *At*TAF4–RST domain, which allowed comparison of αα-hub domains and their interactions. Although the *At*RCD1 TF ligands *At*DREB2A and ANAC013 bound to both TAF4 αα-hub domains, NMR and thermodynamic analyses suggested that only biologically relevant αα-hub–TF pairs have evolved to specificity (57). Moreover, unfolding thermodynamics suggested the existence of a common thermal unfolding state with similar properties in all three αα-hub domains, but with varying temperature sensitivity, suggesting variability in structural adaptability relevant to binding. Taken together, the results showed that not only the flexibility of the TFs ease αα-hub–based protein–protein interactions, but that malleability of the hub domains also contributes to specificity in complex formation, with structure, dynamics, and thermodynamics of binding constituting routes for impacting interactome size.

## Experimental procedures

### Bioinformatics analysis

The domain architectures of *At*RCD1 (Q8RY59), *At*TAF4 (AT5G43130), and *Hs*TAF4 (O00268) were as reported in the Pfam database (59). The interactomes were obtained from the IntAct Molecular Interaction Database (60) selecting for experimentally verified interactions. Multiple sequence alignment of the *At*TAF4–RST, *A*tRCD1–RST, and *Hs*TAF4–TAFH domains were made in ClustalOmega (https://www.ebi.ac.uk/Tools/msa/clustalo/) (61).

### Protein expression and purification

DNA encoding the TAF4–RST_180–254_ domain of *At*TAF4 (AT5G43130) (29, 62) was cloned into pET-11a (Novagen), and the resulting construct verified by sequencing (TAG Copenhagen). The vector was transformed into competent *Escherichia coli* BL21(DE3) cells (Novagen) and subsequently grown in LB medium containing 100 mg ml^−1^ ampicillin at 37 °C under shaking at 150 rpm. Expression of protein was induced with 0.5 mM isopropyl β-d-thiogalactopyranoside at an absorbance of 0.6 to 0.8 at 600 nm. After 3.5 h, cells were harvested by centrifugation (5000*g* for 15 min at 4 °C) and stored at −20 °C. For NMR studies, proteins were expressed as ^15^N, ^13^C-labeled as described (14). For purification of *At*TAF4–RST, cells were resuspended in buffer A (20 mM Tris–HCl, pH 9.0, 20 mM NaCl), lysed by sonication, and the solution clarified by centrifugation at 20,000*g* for 20 min. The supernatant was applied to a 10 ml SOURCE 15S cation exchange column (GE Healthcare) equilibrated with buffer A. A gradient from 0 to 50% buffer B (20 mM Tris–HCl, pH 9.0, and 1 M NaCl) was used for elution.

DNA encoding *Hs*TAF4–TAFH_575–688_ (obtained from TAG Copenhagen) was cloned into pET-15b to produce a fusion protein containing a hexahistidine tag and a tobacco etch virus cleavage sequence positioned at the N terminus. The cells were grown and lysed as described for *At*TAF4–RST, and the supernatant was loaded onto a 2 ml of TALON Metal Affinity resin column (Clontech) equilibrated in 20 mM Tris–HCl, pH 7.0, and 100 mM NaCl. After binding, the resin was washed with the same buffer, and protein was eluted by adding imidazole to 200 mM. Tobacco etch virus protease (produced as described in Ref. (27)) at 1:100 w/w ratio was added, and cleavage was performed overnight and the protein subsequently dialyzed against the purification buffer without imidazole and in the presence of 2 mM DTT and 0.5 mM EDTA. Fractions containing the recombinant protein were further purified on a Superdex 75 10/300 GL column (GE Healthcare) equilibrated with phosphate buffer (20 mM Na_2_HPO_4_/NaH_2_PO_4_, pH 7.4, and 100 mM NaCl). The eluted protein was concentrated using Centricon concentrators (Merck-Millipore) with a 3 kDa cutoff and stored at 4 °C. Final samples were analyzed by SDS-PAGE and MALDI-TOF mass spectrometry. Protein concentrations were calculated using the theoretical absorption coefficients at 280 nm obtained from ProtParam at the EXPASY server.

RCD1–RST_499–572_ was expressed and purified following the protocol as described (14), and *At*ANAC013_254–274_ (AT1G32870) and *At*DREB2A_243–272_ (AT5G05410) were expressed and purified as described by O’Shea *et al.* (27).

### SAXS

All SAXS measurements were carried out at the PETRA III, P12 beamline (DESY Synchrotron), at a working energy of 10 keV. The sample-to-detector distance of the X-rays was 3 m, and the exposure time was optimized to reduce radiation damage (Table S1). Six different concentrations of *At*TAF4–RST_182–254_ were measured (from 1.1 to 8.9 mg ml^−1^) (Table S2). Data from the highest and lowest concentration samples were discarded because of variation in the derived parameters. The samples were in 20 mM Na_2_HPO_4_/NaH_2_PO_4_, pH 7.0, 100 mM NaCl, and 1 mM DTT. The data were calibrated using water at the same temperature and analyzed using the ATSAS program package (https://www.embl-hamburg.de/biosaxs/software.html) (63). The higher concentration (4.2 mg ml^−1^) was used to generate *ab initio* models with DAMAVER and DAMMIF programs from the ATSAS suite (63). The models resulting from 20 independent DAMMIF runs were superimposed using the DAMAVER tool, and the average filtered envelope was superimposed with the NMR structures using SUPCOMB (part of the ATSAS package (63)).

### NMR spectroscopy

NMR data were acquired at 25 °C in 20 mM Na_2_HPO_4_/NaH_2_PO_4_, pH 7.0, 100 mM NaCl, 10% (v/v) D_2_O, 0.02% (w/v) NaN_3_, and 0.7 mM 2,2-dimethyl-2-silapentane-5-sulphonate (DSS) and protein as specified. All spectra used for resonance assignment were recorded on a sample containing 580 μM ^13^C,^15^N-labeled *At*TAF4–RST. For backbone chemical shift assignment, a set of ^1^H,^15^N HSQC, HNCACB, CBCA(CO)NH, HNCO, HN(CA)CO, and (H)N(CA)NNH spectra were recorded on a Bruker AVANCE 600 MHz (^1^H) spectrometer equipped with a cryogenic probe. Side-chain assignments were performed from ^1^H–^13^C HSQC, HCCH-TOCSY, and ^15^N TOCSY–HSQC spectra recorded on a Varian INOVA 800 MHz (^1^H) spectrometer with a room temperature probe. ^15^N NOESY–HSQC and ^13^C NOESY–HSQC spectra were recorded using a mixing time of 150 ms on the Varian INOVA 800 MHz spectrometer. A set of ^1^H,^15^N HSQC, HNCACB, and CBCA(CO)NH spectra were recorded on a Bruker AVANCE 800 MHz (^1^H) spectrometer equipped with a cryogenic probe on a sample containing 200 μM ^13^C,^15^N-labeled DREB2A_243–272_ in complex with 300 μM *At*TAF4–RST. All triple resonance spectra, except NOESY spectra, were recorded with nonuniform sampling at 25% and were reconstructed with quantum multiple-valued decision diagrams (64). All spectra were processed with NMRPipe (https://spin.niddk.nih.gov/bax/software/NMRPipe) and analyzed in CcpNMR analysis (65, 66). Random coil chemical shifts for calculation of secondary ^13^C^α^ chemical shifts were predicted by the webserver available at www.bio.ku.dk/english/research/bms/sbinlab/randomchemicalshifts2 (67).

### Structure calculations

Backbone dihedral angle restraints were calculated using TALOS+, and distance restraints were obtained from ^15^N-NOESY–HSQC and aliphatic and aromatic ^13^C-NOESY–HSQC spectra (68, 69). NOESY peaks were picked manually, whereas automated assignment and initial structure calculations were performed by CYANA (http://www.cyana.org/wiki/index.php/Main_Page) (68). Structure refinement with implicit water solvation potential EEFx (Effective Energy Function for XPLOR-NIH) (70) was performed using XPLOR-NIH resulting in 200 structures, of which the 20 lowest energy structures without significant violations were chosen to represent *At*TAF4–RST. Quality and statistics for the structural ensemble were evaluated with PROCHECK-NMR (71).

### NMR relaxation

*R*_1_, *R*_2_, and ^1^H–^15^N NOE relaxation parameters were determined from spectra recorded on a Bruker AVANCE 750 MHz (^1^H) spectrometer equipped with a cryogenic probe using standard Bruker pulse sequences. Spectra were recorded on a sample containing 480 μM ^15^N-labeled *At*TAF4–RST. Relaxation delays of 20, (3 × 60), 100, 200, 400, (3 × 600), 800, and 1200 ms were used for *R*_1_ and 16.96, (3 × 33.92), 67.84, 101.76, (3 × 135.68), 169.60, 203.52, and 237.44 ms for *R*_2_. A recycle delay of 2.5 s was used in both experiments. For ^1^H–^15^N NOE, two spectra with and without presaturation were recorded in an interleaved manner and with a recycle delay of 5 s. Data analysis was performed in CcpNMR analysis.

### CD spectroscopy

CD spectra were measured using a Jasco 810 spectropolarimeter equipped with a Peltier thermoregulation system. Far-UV CD spectra were recorded between 260 and 190 nm with 0.1 mg ml^−1^ of protein in 20 mM sodium phosphate buffer (Na_2_HPO_4_/NaH_2_PO_4_) at pH 7.4 and 1 mm path length. The scanning speed was 20 nm min^−1^, with data pitch of 0.1 nm. Each spectrum was averaged over 10 scans, and the spectrum of buffer, recorded identically, was subtracted from the protein spectrum. Helicity was calculated from θ_222_ as described (72). For thermal unfolding, the protein concentration was increased to 1 mg ml^−1^, and the samples were in a buffer of 20 mM Na_2_HPO_4_/NaH_2_PO_4,_ pH 7.4, 100 mM NaCl. The signal followed a fixed wavelength of 222 nm in the temperature range of 20 to 90 °C, with data pitch 1 °C and a temperature slope of 1 °C min^−1^. Spectra were also recorded in the presence of increasing urea concentrations from 0 to 8 M. The urea concentration was measured with a Pocket Refractometer (ATAGO Co). Chemical denaturation was monitored by measuring the ellipticity values at 222 nm. Signals above the maximum value of the high-tension voltage, as provided by the spectropolarimeter manufacturer (600 V), were disregarded. Chemical and thermal denaturation curves were fitted as described later.

### Fluorescence spectroscopy

Measurements were performed on the Prometheus NT.48 system (Nanotemper Technologies). Protein samples of 60 μM in phosphate buffer (20 mM Na_2_HPO_4_/NaH_2_PO_4_, pH 7.4, and 100 mM NaCl) and in the presence of different urea concentrations (from 0 to 8 M) were analyzed in Prometheus NT.48 Standard capillaries (Nanotemper Technologies).

### Stability studies

To obtain the Δ*G*_DN_ at 25 °C and the *m* values, the chemical denaturation results measured by CD were fitted to Equation 1:

(1) $$\begin{array}{l} y_{\left(c\right)}=\frac{y_{N}\left(c\right)+y_{D}\left(c\right)\exp\frac{\Delta G-mc}{RT}}{1+exp\frac{\Delta G-mc}{RT}} \end{array}$$

where $y_{\left(c\right)}$ is the optical property at *c* (M) of denaturant; $y_{N}\left(c\right)$ and $y_{D}\left(c\right)$ are the optical properties of the native and the denatured protein molecules at *c* (M), respectively, and *R* is the gas constant.

Thermal denaturation was analyzed using the nonlinear least square fitting:

(2) $$\begin{array}{l} y_{\left(T\right)}=\frac{y_{N}\left(T\right)+y_{D}\left(T\right)\exp\frac{\Delta H_{vH}\left(1-\frac{T}{T_{\text{m}}}\right)}{RT}}{1+\exp\frac{\Delta H_{vH}\left(1-\frac{T}{T_{\text{m}}}\right)}{RT}} \end{array}$$

where $y_{\left(T\right)}$ is the optical property at *T* (K) of denaturant; $y_{N}\left(T\right)$ and $y_{D}\left(T\right)$ are the optical properties of the native and denatured protein molecules at *T* (K), respectively, and *R* is the gas constant. The midpoint of denaturation (*T*_m_) and *ΔH*_vH_ were calculated for each protein. For the stability studies using CD spectroscopy, the spectra were analyzed using GraphPad Prism 9.0 (GraphPad Software, Inc).

The curves obtained from the fluorescence experiments were fitted to a two-dimensional model based on a two-step denaturation using Equation 3:

(3) $$\begin{array}{l} y_{\left(T,\;\left[x\right]\right)}=\frac{y_{N}\left(T\right)+y_{D}\left(T\right)\exp\frac{\Delta H_{\text{m}}\left(1-\frac{T}{T_{\text{m}}}\right)+\Delta C_{\text{p}}(T-T_{\text{m}}-T\;\ln\left(\frac{T}{T_{\text{m}}})\right)-\left[x\right](m+m_{1}T+m_{2}T^{2})}{RT}}{1+\exp\frac{\Delta H_{\text{m}}\left(1-\frac{T}{T_{\text{m}}}\right)+\Delta C_{\text{p}}(T-T_{\text{m}}-T\;\ln\left(\frac{T}{T_{\text{m}}})\right)-\left[x\right](m+m_{1}T+m_{2}T^{2})}{RT}} \end{array}$$

Equation 3 represents the global fit that consider both thermal and chemical denaturation, where *ΔH*_m_ is the enthalpy change at the *T*_m_, Δ*C*_p_ is the heat capacity change, and *m*, *m*_1_, and *m*_2_ describe the *m* value at changing of denaturant concentration.

$y_{N}\left(T\right)$ and $y_{D}\left(T\right)\;$ describe the pretransition baseline and the post-transition baseline, respectively:

(4) $$\begin{array}{l} y_{N}\left(T\right)=a_{N}+b_{N}T+c_{N}T^{2} \end{array}$$

(5) $$\begin{array}{l} y_{D}\left(T\right)=a_{D}+b_{D}T+c_{D}T^{2} \end{array}$$

where *a*_*N*_, *b*_*N*_, *c*_*N*_, *a*_*D*_, *b*_*D*_, and *c*_*D*_ are temperature-independent coefficients. Pretransition and post-transition baselines of the denaturation experiments followed by CD spectroscopy were included in the fit but omitted from Fig S3. These baselines may be caused by solvent effects on the far-UV CD signal of the domains in the folded (pretransition) or unfolded (post-transition) states, respectively.

Gibbs free-energy change of protein unfolding was estimated with Equation 3, with values of Δ*H*_m_, Δ*C*_p_, *T*_m_, and *m*, *m*_1_, and *m*_2_.

(6) $$\begin{array}{l} \Delta G_{\left(T,\left[x\right]\right)}=\Delta H_{\text{m}}\left(1-\frac{T}{T_{\text{m}}}\right)+\Delta C_{\text{p}}\left(T-T_{\text{m}}-T\;\ln\left(\frac{T}{T_{\text{m}}}\right)\right)-\left[x\right]\left(m+m_{1}T+m_{2}T^{2}\right) \end{array}$$

The global analysis of temperature and solvent denaturation was performed according to Ref. (44).

The *C*_m_ value was determined by

(7) $$\begin{array}{l} C_{\text{m}}=\frac{\Delta G}{m}. \end{array}$$

### NMR titration experiments

The interaction between *At*TAF4–RST and *At*DREB2A_243–272_ was investigated through a series of ^1^H,^15^N HSQC spectra recorded on samples containing 100 μM *At*TAF4–RST in 20 mM Na_2_HPO_4_/NaH_2_PO_4_, pH 7.0, 100 mM NaCl, 10% (v/v) D_2_O, 0.02% (w/v) NaN_3_, and 0.7 mM DSS buffer and varying concentrations of DREB2A_243–272_ from 0 to 200 μM. Amide chemical shift perturbations between free and bound states were quantified using the weighted Euclidean distance (73):

(8) Δδ15N,HN(ppm)=(ΔδH1)2+(0.154∗ΔδN15)2

### NMR temperature experiments

The chemical shifts of *At*TAF4–RST at different temperatures were investigated through a series of ^1^H,^15^N HSQC spectra recorded on samples containing 100 μM ^15^N-labeled *At*TAF4–RST or *At*RCD1–RST in 20 mM Na_2_HPO_4_/NaH_2_PO_4_, pH 7.0, 100 mM NaCl, 10% (v/v) D_2_O, 0.02% (w/v) NaN_3_, and 125 μM DSS buffer, at 25, 30, 35, 40, 45, 50, and 55 °C.

### ITC

ITC experiments were performed in a MicroCal ITC_200_ microcalorimeter (GE Healthcare). Protein samples at the concentration of 27 μM in the sample cell and 277 μM in the syringe were dialyzed against 50 mM Hepes buffer, pH 7.4, and 100 mM NaCl. ITC data were analyzed using an Origin 7 software package (MicroCal) and fitting to a one set of sites binding model. At least two experiments were performed for each interaction.

## Data availability

Chemical shifts and NOESY data for *At*TAF4–RST have been deposited in the Biological Magnetic Resonance Bank, www.bmrb.wisc.edu.org under ID code 34557. Atomic coordinates have been deposited in the Protein Data Bank, www.pdb.org, under ID code 7AC1.

## Supporting information

This article contains supporting information (38).

## Conflict of interest

The authors declare that they have no conflicts of interest with the contents of this article.

## References

[1] Ma J., Ptashne M. (1987). A new class of yeast transcriptional activators. Cell.

[2] Kundu T.K., Palhan V.B., Wang Z., An W., Cole P.A., Roeder R.G. (2000). Activator-dependent transcription from chromatin *in vitro* involving targeted histone acetylation by p300. Mol. Cell.

[3] Reeves W.M., Hahn S. (2005). Targets of the Gal4 transcription activator in functional transcription complexes. Mol. Cell Biol..

[4] Berlow R.B., Dyson H.J., Wright P.E. (2017). Hypersensitive termination of the hypoxic response by a disordered protein switch. Nature.

[5] Ptashne M., Gann A. (1997). Transcriptional activation by recruitment. Nature.

[6] Erkina T.Y., Erkine A.M. (2016). Nucleosome distortion as a possible mechanism of transcription activation domain function. Epigenetics and Chromatin.

[7] Sigler P.B. (1988). Transcriptional activation. Acid blobs and negative noodles. Nature.

[8] Staby L., O’Shea C., Willemoës M., Theisen F., Kragelund B.B., Skriver K. (2017). Eukaryotic transcription factors: Paradigms of protein intrinsic disorder. Biochem. J..

[9] Warfield L., Tuttle L.M., Pacheco D., Klevit R.E., Hahn S. (2014). A sequence-specific transcription activator motif and powerful synthetic variants that bind Mediator using a fuzzy protein interface. Proc. Natl. Acad. Sci. U. S. A..

[10] Tuttle L.M., Pacheco D., Warfield L., Luo J., Ranish J., Hahn S., Klevit R.E. (2018). Gcn4-Mediator specificity is mediated by a large and dynamic fuzzy protein-protein complex. Cell Rep..

[11] Ravarani C.N., Erkina T.Y., De Baets G., Dudman D.C., Erkine A.M., Babu M.M. (2018). High-throughput discovery of functional disordered regions: Investigation of transactivation domains. Mol. Syst. Biol..

[12] Henley M.J., Linhares B.M., Morgan B.S., Cierpicki T., Fierke C.A., Mapp A.K. (2020). Unexpected specificity within dynamic transcriptional protein–protein complexes. Proc. Natl. Acad. Sci. U. S. A..

[13] Bugge K., Staby L., Salladini E., Falbe-Hansen R.G., Kragelund B.B., Skriver K. (2021). αα-Hub domains and intrinsically disordered proteins: A decisive combo. J. Biol. Chem..

[14] Bugge K., Staby L., Kemplen K.R., O’Shea C., Bendsen S.K., Jensen M.K., Olsen J.G., Skriver K., Kragelund B.B. (2018). Structure of radical-induced cell Death1 hub domain reveals a common αα-scaffold for disorder in transcriptional networks. Structure.

[15] Staby L., Bugge K., Falbe-Hansen R.G., Salladini E., Skriver K., Kragelund B.B. (2021). Connecting the αα-hubs: Same fold, disordered ligands, new functions. Cell Commun. Signal..

[16] Dyson H.J., Wright P.E. (2016). Role of intrinsic protein disorder in the function and interactions of the transcriptional coactivators CREB-binding protein (CBP) and p300. J. Biol. Chem..

[17] Adams G.E., Chandru A., Cowley S.M. (2018). Co-repressor, co-activator and general transcription factor: The many faces of the Sin3 histone deacetylase (HDAC) complex. Biochem. J..

[18] Jaspers P., Brosché M., Overmyer K., Kangasjär J. (2010). The transcription factor interacting protein RCD1 contains a novel conserved domain. Plant Signal. Behav..

[19] Jaspers P., Blomster T., Brosché M., Salojärvi J., Ahlfors R., Vainonen J.P.P., Reddy R.A.A., Immink R., Angenent G., Turck F., Overmyer K., Kangasjärvi J. (2009). Unequally redundant RCD1 and SRO1 mediate stress and developmental responses and interact with transcription factors. Plant J..

[20] Brosché M., Blomster T., Salojärvi J., Cui F., Sipari N., Leppälä J., Lamminmäki A., Tomai G., Narayanasamy S., Reddy R.A., Keinänen M., Overmyer K., Kangasjärvi J. (2014). Transcriptomics and functional genomics of ROS-induced cell death regulation by radical-induced cell Death1. PLoS Genet..

[21] Wirthmueller L., Asai S., Rallapalli G., Sklenar J., Fabro G., Kim D.S., Lintermann R., Jaspers P., Wrzaczek M., Kangasjärvi J., MacLean D., Menke F.L.H., Banfield M.J., Jones J.D.G. (2018). Arabidopsis downy mildew effector HaRxL106 suppresses plant immunity by binding to radical-induced cell Death1. New Phytol..

[22] Kragelund B.B.B., Jensen M.K., Skriver K. (2012). Order by disorder in plant signaling. Trends Plant Sci..

[23] Vainonen J.P., Jaspers P., Wrzaczek M., Lamminmäki A., Reddy R.A., Vaahtera L., Brosché M., Kangasjärvi J. (2012). RCD1-DREB2A interaction in leaf senescence and stress responses in Arabidopsis thaliana. Biochem. J..

[24] Shapiguzov A., Vainonen J.P., Hunter K., Tossavainen H., Tiwari A., Järvi S., Hellman M., Aarabi F., Alseekh S., Wybouw B., van der Kelen K., Nikkanen L., Krasensky-Wrzaczek J., Sipari N., Keinänen M. (2019). Arabidopsis RCD1 coordinates chloroplast and mitochondrial functions through interaction with ANAC transcription factors. eLife.

[25] De Clercq I., Vermeirssen V., Van Aken O., Vandepoele K., Murcha M.W., Law S.R., Inzé A., Ng S., Ivanova A., Rombaut D., van de Cotte B., Jaspers P., Van de Peer Y., Kangasjärvi J., Whelan J. (2013). The membrane-bound NAC transcription factor ANAC013 functions in mitochondrial retrograde regulation of the oxidative stress response in Arabidopsis. Plant Cell.

[26] O’Shea C., Kryger M., Stender E.G.P.P., Kragelund B.B., Willemoes M., Skriver K., O’Shea C., Kryger M., Stender E.G.P.P., Kragelund B.B., Willemoës M., Skriver K. (2015). Protein intrinsic disorder in Arabidopsis NAC transcription factors: Transcriptional activation by ANAC013 and ANAC046 and their interactions with RCD1. Biochem. J..

[27] O’Shea C., Staby L., Bendsen S.K., Tidemand F.G., Redsted A., Willemoës M., Kragelund B.B., Skriver K. (2017). Structures and short linear motif of disordered transcription factor regions provide clues to the interactome of the cellular hub protein radical-induced cell Death1. J. Biol. Chem..

[28] Christensen L.F., Staby L., Bugge K., O’Shea C., Kragelund B.B., Skriver K. (2019). Evolutionary conservation of the intrinsic disorder-based radical-induced cell Death1 hub interactome. Sci. Rep..

[29] Lago C., Clerici E., Mizzi L., Colombo L., Kater M.M. (2004). TBP-associated factors in Arabidopsis. Gene.

[30] Lawrence E.J., Gao H., Tock A.J., Lambing C., Blackwell A.R., Feng X., Henderson I.R. (2019). Natural variation in TBP-associated factor 4b controls meiotic crossover and germline transcription in Arabidopsis. Curr. Biol..

[31] Wright K.J., Marr M.T., Tjian R. (2006). TAF4 nucleates a core subcomplex of TFIID and mediates activated transcription from a TATA-less promoter. Proc. Natl. Acad. Sci. U. S. A..

[32] Marr M.T. (2009). TAF4 takes flight. Proc. Natl. Acad. Sci. U. S. A..

[33] Lawit S.J., O’Grady K., Gurley W.B., Czarnecka-Verner E. (2007). Yeast two-hybrid map of Arabidopsis TFIID. Plant Mol. Biol..

[34] Chen W.Y., Zhang J., Geng H., Du Z., Nakadai T., Roeder R.G. (2013). A TAF4 coactivator function for E proteins that involves enhanced TFIID binding. Genes Dev..

[35] Wang X., Truckses D.M., Takada S., Matsumura T., Tanese N., Jacobson R.H. (2007). Conserved region I of human coactivator TAF4 binds to a short hydrophobic motif present in transcriptional regulators. Proc. Natl. Acad. Sci. U. S. A..

[36] Efimov A.V. (1991). Structure of α-α-hairpins with short connections. Protein Eng. Des. Selection.

[37] Park S., Chen W., Cierpicki T., Tonelli M., Cai X., Speck N.A., Bushweller J.H. (2009). Structure of the AML1-ETO eTAFH domain–HEB peptide complex and its contribution to AML1-ETO activity. Blood.

[38] Staby L., Due A.D., Kunze M.B.A., Jørgensen M.L.M., Skriver K., Kragelund B.B. (2021). Flanking disorder of the folded αα-hub domain from radical induced cell Death1 affects transcription factor binding by ensemble redistribution. J. Mol. Biol..

[39] Theisen F.F., Staby L., Tidemand F.G., O’Shea C., Prestel A., Willemoës M., Kragelund B.B., Skriver K. (2021). Quantification of conformational entropy unravels effect of disordered flanking region in coupled folding and binding. J. Am. Chem. Soc..

[40] Kneller J.M., Lu M., Bracken C. (2002). An effective method for the discrimination of motional anisotropy and chemical exchange. J. Am. Chem. Soc..

[41] Berlow R.B., Martinez-Yamout M.A., Dyson H.J., Wright P.E. (2019). Role of backbone dynamics in modulating the interactions of disordered ligands with the TAZ1 domain of the CREB-binding protein. Biochemistry.

[42] Alhindi T., Zhang Z., Ruelens P., Coenen H., Degroote H., Iraci N., Geuten K. (2017). Protein interaction evolution from promiscuity to specificity with reduced flexibility in an increasingly complex network. Sci. Rep..

[43] Shirdel S.A., Khalifeh K. (2019). Thermodynamics of protein folding: Methodology, data analysis and interpretation of data. Eur. Biophys. J..

[44] Hamborg L., Horsted E.W., Johansson K.E., Willemoës M., Lindorff-Larsen K., Teilum K. (2020). Global analysis of protein stability by temperature and chemical denaturation. Anal. Biochem..

[45] Myers J.K., Nick Pace C., Martin Scholtz J. (1995). Denaturant m values and heat capacity changes: Relation to changes in accessible surface areas of protein unfolding. Protein Sci..

[46] Nettels D., Müller-Späth S., Küster F., Hofmann H., Haenni D., Rüegger S., Reymond L., Hoffmann A., Kubelka J., Heinz B., Gast K., Best R.B., Schuler B. (2009). Single-molecule spectroscopy of the temperature-induced collapse of unfolded proteins. Proc. Natl. Acad. Sci. U. S. A..

[47] Narayan A., Bhattacharjee K., Naganathan A.N. (2019). Thermally *versus* chemically denatured protein states. Biochemistry.

[48] Jaspers P., Overmyer K., Wrzaczek M., Vainonen J.P., Blomster T., Salojärvi J., Reddy R.A., Kangasjärvi J. (2010). The RST and PARP-like domain containing SRO protein family: Analysis of protein structure, function and conservation in land plants. BMC Genomics.

[49] Sanborn A.L., Yeh B.T., Feigerle J.T., Hao C. v, Townshend R.J., Lieberman Aiden E., Dror R.O., Kornberg R.D. (2021). Simple biochemical features underlie transcriptional activation domain diversity and dynamic, fuzzy binding to mediator. eLife.

[50] Ferreira R.M., Rybarczyk-Filho J.L., Dalmolin R.J.S., Castro M.A.A., Moreira J.C.F., Brunnet L.G., de Almeida R.M.C. (2013). Preferential duplication of intermodular hub genes: An evolutionary signature in eukaryotes genome networks. PLoS One.

[51] Prabhu N.V., Sharp K.A. (2005). Heat capacity in proteins. Annu. Rev. Phys. Chem..

[52] Vainonen J.P., Shapiguzov A., Krasensky-Wrzaczek J., De Masi R., Gossens R., Danciu I., Battchikova N., Jonak C., Wirthmueller L., Wrzaczek M., Kangasjärvi J. (2020). Arabidopsis poly(ADP-ribose)-binding protein RCD1 interacts with photoregulatory protein kinases in nuclear bodies. bioRxiv.

[53] Gianni S., Jemth P. (2019). Affinity *versus* specificity in coupled binding and folding reactions. Protein Eng. Des. Selection.

[54] Had Zi S., Loris R., Lah J. (2021). The sequence-ensemble relationship in fuzzy protein complexes. Proc. Natl. Acad. Sci. U. S. A..

[55] Lawrence C.W., Kumar S., Noid W.G., Showalter S.A. (2014). Role of ordered proteins in the folding-upon-binding of intrinsically disordered proteins. J. Phys. Chem. Lett..

[56] Borgia A., Borgia M.B., Bugge K., Kissling V.M., Heidarsson P.O., Fernandes C.B., Sottini A., Soranno A., Buholzer K.J., Nettels D., Kragelund B.B., Best R.B., Schuler B. (2018). Extreme disorder in an ultrahigh-affinity protein complex. Nature.

[57] Jemth P., Karlsson E., Vögeli B., Guzovsky B., Andersson E., Hultqvist G., Dogan J., Güntert P., Riek R., Chi C.N. (2018). Structure and dynamics conspire in the evolution of affinity between intrinsically disordered proteins. Sci. Adv..

[58] Ahlfors R., Lång S., Overmyer K., Jaspers P., Brosché M., Tauriainen A., Kollist H., Tuominen H., Belles-Boix E., Piippo M., Inzé D., Palva E.T., Kangasjärvi J. (2004). Arabidopsis radical-induced cell Death1 belongs to the WWE protein–protein interaction domain protein family and modulates abscisic acid, ethylene, and methyl jasmonate responses. The Plant Cell.

[59] El-Gebali S., Mistry J., Bateman A., Eddy S.R., Luciani A., Potter S.C., Qureshi M., Richardson L.J., Salazar G.A., Smart A., Sonnhammer E.L.L., Hirsh L., Paladin L., Piovesan D., Tosatto S.C.E. (2019). The Pfam protein families database in 2019. Nucl. Acids Res..

[60] Orchard S., Ammari M., Aranda B., Breuza L., Briganti L., Broackes-Carter F., Campbell N.H., Chavali G., Chen C., Del-Toro N., Duesbury M., Dumousseau M., Galeota E., Hinz U., Iannuccelli M. (2014). The MIntAct project - IntAct as a common curation platform for 11 molecular interaction databases. Nucl. Acids Res..

[61] Sievers F., Wilm A., Dineen D., Gibson T.J., Karplus K., Li W., Lopez R., McWilliam H., Remmert M., Söding J., Thompson J.D., Higgins D.G. (2011). Fast, scalable generation of high-quality protein multiple sequence alignments using Clustal Omega. Mol. Syst. Biol..

[62] Wang Y., Zhang W.Z., Song L.F., Zou J.J., Su Z., Wu W.H. (2008). Transcriptome analyses show changes in gene expression to accompany pollen germination and tube growth in Arabidopsis. Plant Physiol..

[63] Franke D., Petoukhov M.V., Konarev P.V., Panjkovich A., Tuukkanen A., Mertens H.D.T., Kikhney A.G., Hajizadeh N.R., Franklin J.M., Jeffries C.M., Svergun D.I. (2017). Atsas 2.8: A comprehensive data analysis suite for small-angle scattering from macromolecular solutions. J. Appl. Crystallogr..

[64] Orekhov V.Y., Jaravine V.A. (2011). Analysis of non-uniformly sampled spectra with multi-dimensional decomposition. Prog. Nucl. Magn. Reson. Spectrosc..

[65] Delaglio F., Grzesiek S., Vuister G.W., Zhu G., Pfeifer J., Bax A. (1995). NMRPipe: A multidimensional spectral processing system based on UNIX pipes. J. Biomol. NMR.

[66] Vranken W.F., Boucher W., Stevens T.J., Fogh R.H., Pajon A., Llinas M., Ulrich E.L., Markley J.L., Ionides J., Laue E.D. (2005). The CCPN data model for NMR spectroscopy: Development of a software pipeline. Proteins: Struct. Funct. Genet..

[67] Kjaergaard M., Poulsen F.M. (2011). Sequence correction of random coil chemical shifts: Correlation between neighbor correction factors and changes in the Ramachandran distribution. J. Biomol. NMR.

[68] Güntert P. (2004). Automated NMR structure calculation with CYANA. Methods Mol. Biol. (Clifton, N.J.).

[69] Shen Y., Delaglio F., Cornilescu G., Bax A. (2009). TALOS+: A hybrid method for predicting protein backbone torsion angles from NMR chemical shifts. J. Biomol. NMR.

[70] Tian Y., Schwieters C.D., Opella S.J., Marassi F.M. (2014). A practical implicit solvent potential for NMR structure calculation. J. Magn. Reson..

[71] Laskowski R.A., Rullmann J.A.C., MacArthur M.W., Kaptein R., Thornton J.M. (1996). AQUA and PROCHECK-NMR: Programs for checking the quality of protein structures solved by NMR. J. Biomol. NMR.

[72] Rohl C.A., Baldwin R.L. (1997). Comparison of NH exchange and circular dichroism as techniques for measuring the parameters of the Helix−Coil transition in peptides. Biochemistry.

[73] Mulder F.A.A., Schipper D., Bott R., Boelens R. (1999). Altered flexibility in the substrate-binding site of related native and engineered high-alkaline Bacillus subtilisins. J. Mol. Biol..

[74] Jurrus E., Engel D., Star K., Monson K., Brandi J., Felberg L.E., Brookes D.H., Wilson L., Chen J., Liles K., Chun M., Li P., Gohara D.W., Dolinsky T., Konecny R. (2018). Improvements to the APBS biomolecular solvation software suite. Protein Sci..

