# Supplementary material for: αα-hub coregulator structure and flexibility determine transcription factor binding and selection in regulatory interactomes
Source: J Biol Chem. 2022 Apr 20;298(6):101963. doi: 10.1016/j.jbc.2022.101963 (PMC9127584; doi:10.1016/j.jbc.2022.101963)
Supplement: Supplemental Tables S1–S4 and Figures S1–S6 [file mmc1.pdf]

Supporting Information

αα-hub domains: from structure, stability and binding to selection in transcriptional interactomes

Frederik Friis Theisen[1#], Edoardo Salladini[1#], Rikke Davidsen[1], Christina Jo Rasmussen[1], Lasse Staby[12], Birthe B. Kragelund[12]* and Karen Skriver[1]*

[1] REPIN and the Linderstrøm-Lang Centre for Protein Science, Department of Biology, University of Copenhagen, Copenhagen, DK-2200, Denmark

[2] Structural Biology and NMR Laboratory, Department of Biology, University of Copenhagen, Copenhagen, Denmark

# These authors contributed equally to this work

* Correspondence: Birthe B. Kragelund, bbk@bio.ku.dk; Karen Skriver, kskriver@bio.ku.dk

Running title: *αα-hubs: correlating structure, stability and interactome*

List of contents:

S-2: Table S1 SAXS data collection and derived parameters for *At*TAF4-RST

S-2: Table S2 R_g (from Guinier) and D_{max} for the *At*TAF4-RST domain at various protein concentrations

S-3: Table S3 NMR refinement statistics for *At*TAF4-RST

S-3: Table S4 Parameters from denaturation experiments

S-4: Figure S1 SAXS studies of *At*TAF4-RST

S-5: Figure S2 Dynamics of *At*TAF4-RST

S-6: Figure S3 Stabilities of the *At*RCD1-RST, *At*TAF4-RST and *Hs*TAF4-TAFH domains

S-7: Figure S4 HSQC spectra of thermal unfolding of RST from *At*TAF4 and *At*RCD1

S-8: Figure S5 ITC measurements at different temperatures

S-9: Figure S6 HSQC spectra of free and *At*TAF4-RST bound DREB2A

S-9: References

TABLE S1

SAXS data collection and derived parameters for *At*TAF4-RST

**Data-collection parameters**	
Data source and instrument (beamline)	Hamburg (P12)
Instrument (detector)	PILATUS 6M
Beam geometry (mm^2)	0.2 x 0.12
Wavelength (Å)	1.239
Detector distance (m)	3
s range (nm^{-1})	0.025 - 7.317
Exposure time (sec)	0.5 (50 x 0.01)
Concentration range (mg ml^{-1})	1.12 – 8.93
Temperature (K)	293
**Structural parameters**	
$I(0)$ (cm^{-1}) [from P(r) of normalized samples]	13.8 ± 0.1
Rg (Å) [from P(r)]	15.4 ± 0.4
$I(0)$ (cm^{-1}) [form Guinier approximation of normalized samples]	13.7 ± 0.1
R_g (Å) [from Guinier approximation]	15.4 ± 0.1
D_{max} (Å)	45.0 ± 1.2
**Molecular-mass determination**	
Molecular mass M_r (kDa) [from *water calibration*]	8.56 ± 0.23
Calculated monomeric M_r (kDa) [from *sequence*]*	8.65
**Software employed**	
Primary data reduction	PRIMUS
Data processing	PRIMUS
Ab initio analysis	DAMMIF
Validation and averaging	DAMAVER
Computation of model intensities	CRYSOL

* Obtained using the ProtParam tool of the Expasy server (https://web.expasy.org/protparam/).

TABLE S2

R_g and D_{max} for the *At*TAF4-RST domain at different concentrations

[TAF4] (g L^{-1})	MW (Guinier) (kDa)	R_g (Guinier) (Å)	R_g (P(r)) (Å)	D_{max} (Å)
1.12	7.3 ± 0.7	15.0 ± 0.1	15.1	40.9
2.14	8.5 ± 0.2	15.3 ± 0.1	15.2	43.6
2.59	8.8 ± 0.1	15.3 ± 0.1	15.2	44.8
3.65	9.1 ± 0.1	15.7 ± 0.4	15.3	44.0
4.20	8.6 ± 0.5	15.5 ± 0.1	15.4	45.3
8.93	8.5 ± 1.3	15.8 ± 0.1	15.9	51.3

[*] The expected MW in kDa is 8.9.

TABLE S3

NMR refinement statistics for *At*TAF4-RST

	*At*TAF4-RST
**NMR distance restraints and dihedral constraints**	
Distance restraints	
Total NOEs	1248
Intra-residue	285
Inter-residue	963
Sequential ($\|i - j\| = 1$)	307
Medium-range ($\|i - j\| \leq 5$)	362
Long-range ($\|i - j\| > 5$)	294
Hydrogen bonds	0
Total dihedral angle constraints	116
ϕ	58
ψ	58
**Structure statistics**	
Violations (mean ± sd)	
Distance constraints (Å)	0.025 ± 0.002
Dihedral angle constraints (°)	0.213 ± 0.105
Max. dihedral angle violations (°)	2.41
Max. distance constraint violation (Å)	0.431
Deviations from idealized geometry	
Bond lengths (Å)	0.006 ± 0.000
Bond angles (°)	0.852 ± 0.006
Impropers (°)	0.494 ± 0.011
Average pairwise r.m.s. deviation (Å)	
Heavy (193-250)	1.07 ± 0.14
Backbone (193-250)	0.25 ± 0.07
Ramachandran plot statistics (%)	
Residues in most favored regions	99.2
Residues in additionally allowed regions	0.8
Residues in generously allowed regions	0.0
Residues in disallowed regions	0.0

TABLE S4

Parameters from denaturation experiments

	$\Delta H_{vH}/\Delta H_{m}$	*Cm* (M) (CD chemical denaturation)	*Cm* (M) (Two-dimensional global analysis)
*At*RCD1-RST	0.5	2.6 ± 0.2	2.59 ± 0.01
*At*TAF4-RST	0.4	1.9 ± 0.6	1.74 ± 0.09
*Hs*TAF4-TAFH	0.3	2.4 ± 0.1	3.18 ± 0.03

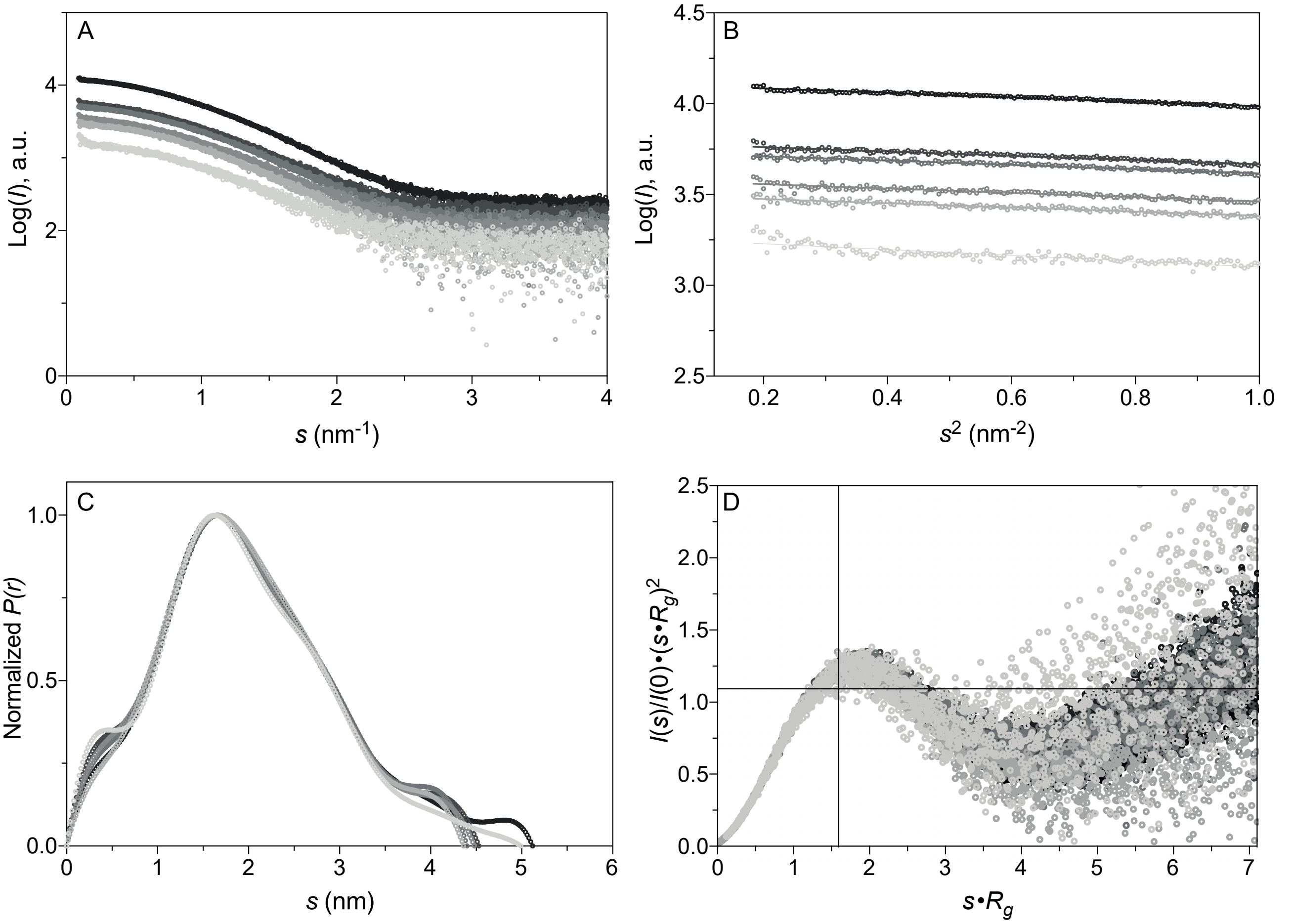

Figure S1. SAXS studies of *At*TAF4-RST. (**A**) SAXS scattering curves of *At*TAF4-RST at different concentrations from light gray (1.12 g/L) to black (8.93 g/L). Parameters from the experiments are presented in Table S1. (**B**) Guinier plots and (**C**) normalized pair distance distribution, *P(r)*. (**D**) Dimensionless Kratky plot representations of the scattering data at different concentrations.

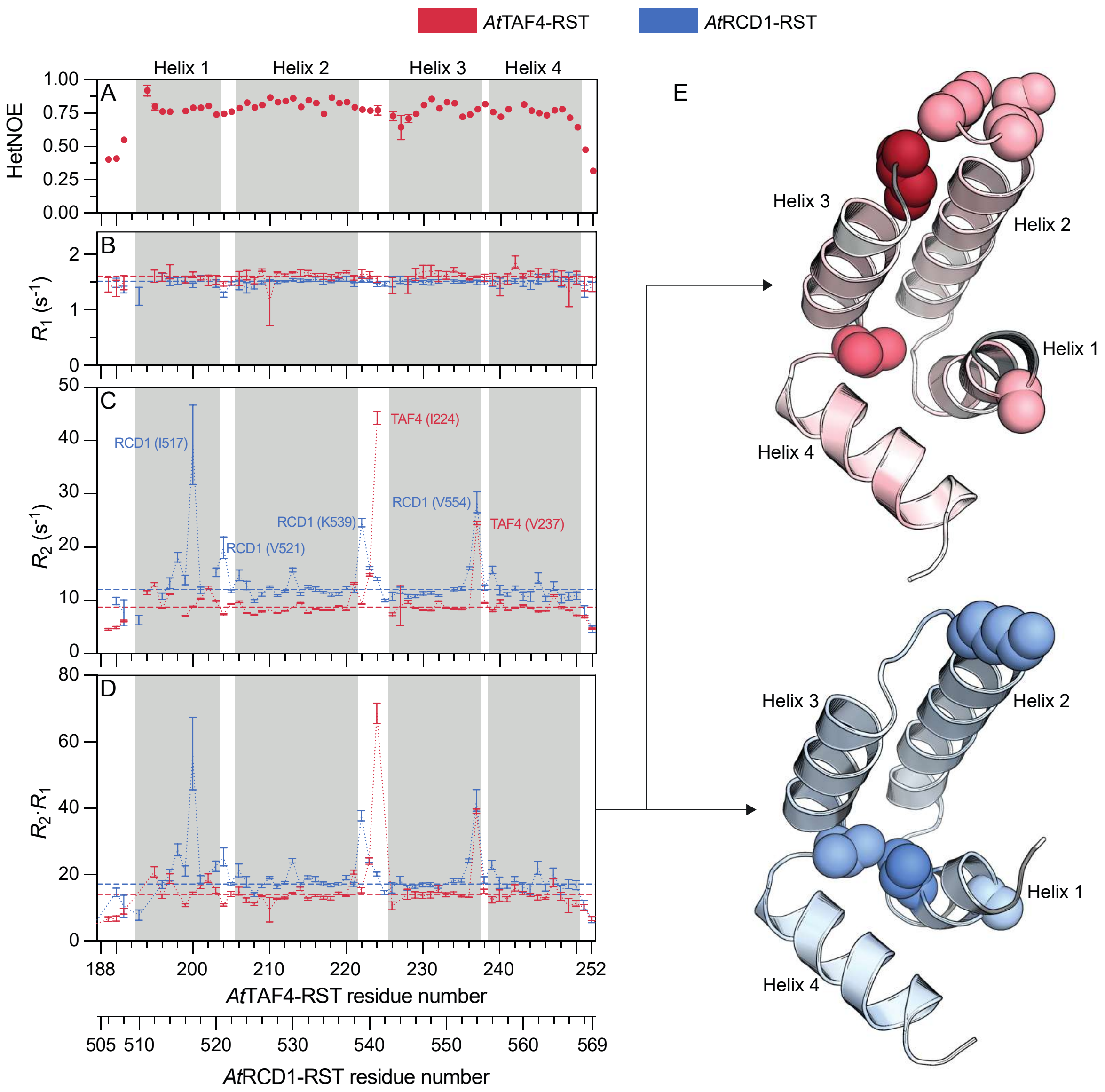

Figure S2. Dynamics of *At*TAF4-RST compared to *At*RCD1-RST. (**A**) ^{1}H-^{15}N HetNOEs for *At*TAF4-RST. (**B**) ^{15}N longitudinal relaxation rates for *At*TAF4-RST (red) and *At*RCD1-RST (blue) (from (1)), the dashed lines indicate the trimmed mean value of residues in secondary structures. (**C**) Transverse relaxation rates. (**D**) Relaxation rate products. Grey background indicates helical structure in *At*TAF4-RST. (**E**) Relaxation rate products highlighted on the structures of the two RST domains. Residues drawn as spheres differ significantly based on a mean + 1 standard deviation criteria.

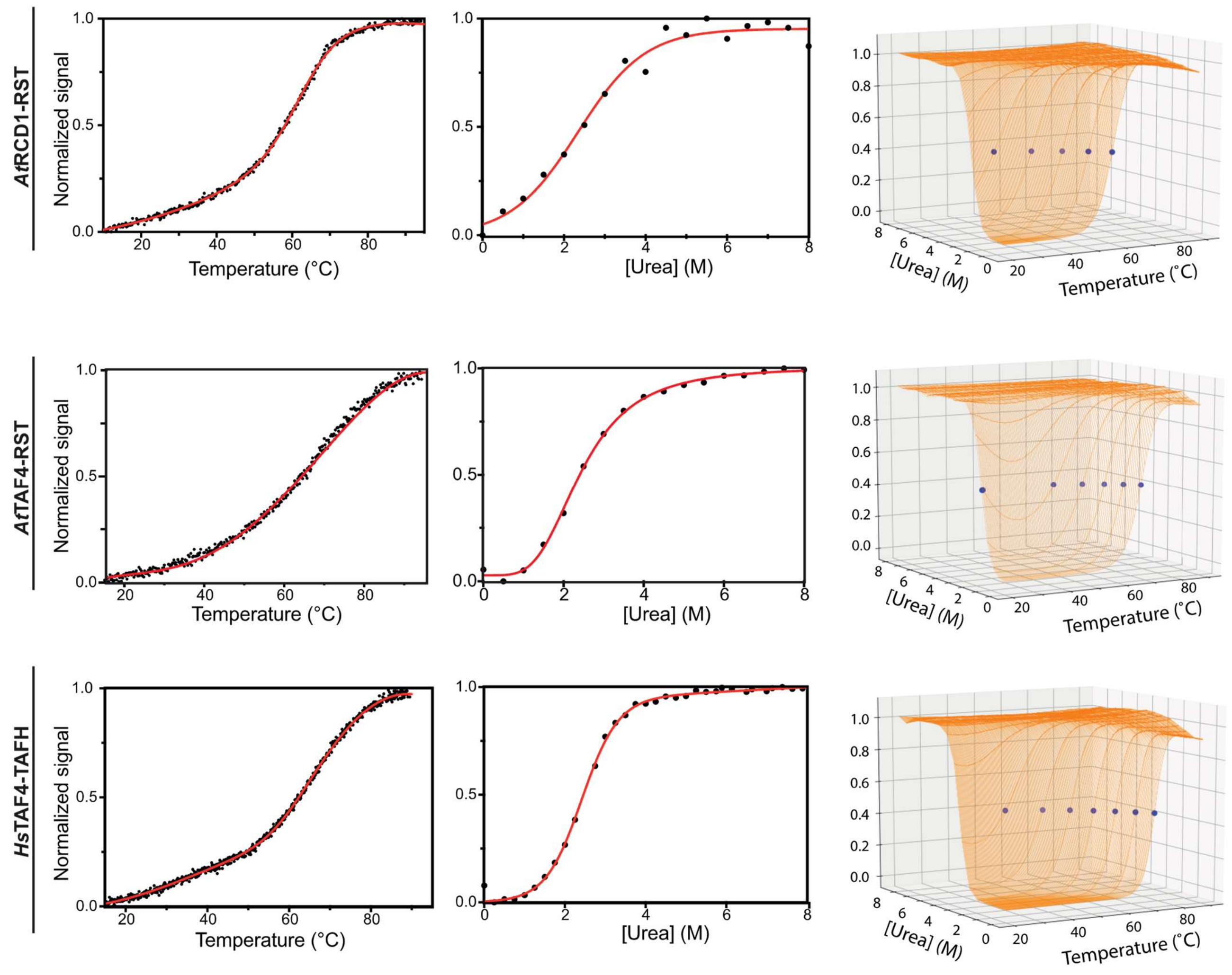

Figure S3. Stability measurements of the *At*RCD1-RST, *At*TAF4-RST and *Hs*TAF4-TAFH domains. Normalized signal from CD spectroscopy (222 nm) of 0.1 mg ml^{-1} protein domain in 20 mM sodium phosphate, pH 7.4, as in function of temperature (left) and urea concentration (middle), or from fluorescence detected unfolding using by two-dimensional global analysis (right) of the domains recorded in sodium phosphate buffer, pH 7.4 at 60 µM protein concentration. The blue circles correspond to the transition point of the curves.

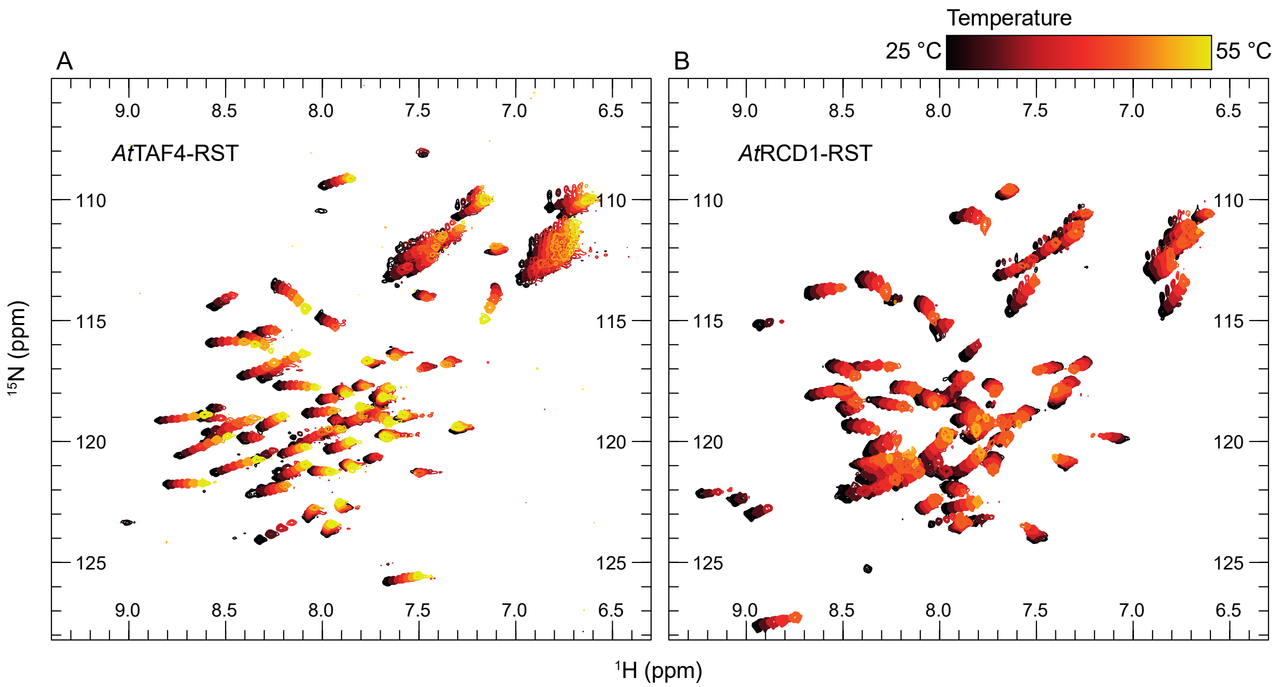

Figure S4. HSQC spectra of thermal unfolding of RST from *At*TAF4 and *At*RCD1. ^{15}N,H^{N} HSQC spectra of ^{15}N-labeled *At*TAF4-RST (**A**) and *At*RCD1-RST (**B**) recorded in 5 °C intervals from 25 °C (black) to 55 °C (yellow). No *At*RCD1-RST peaks could be resolved at 55 °C.

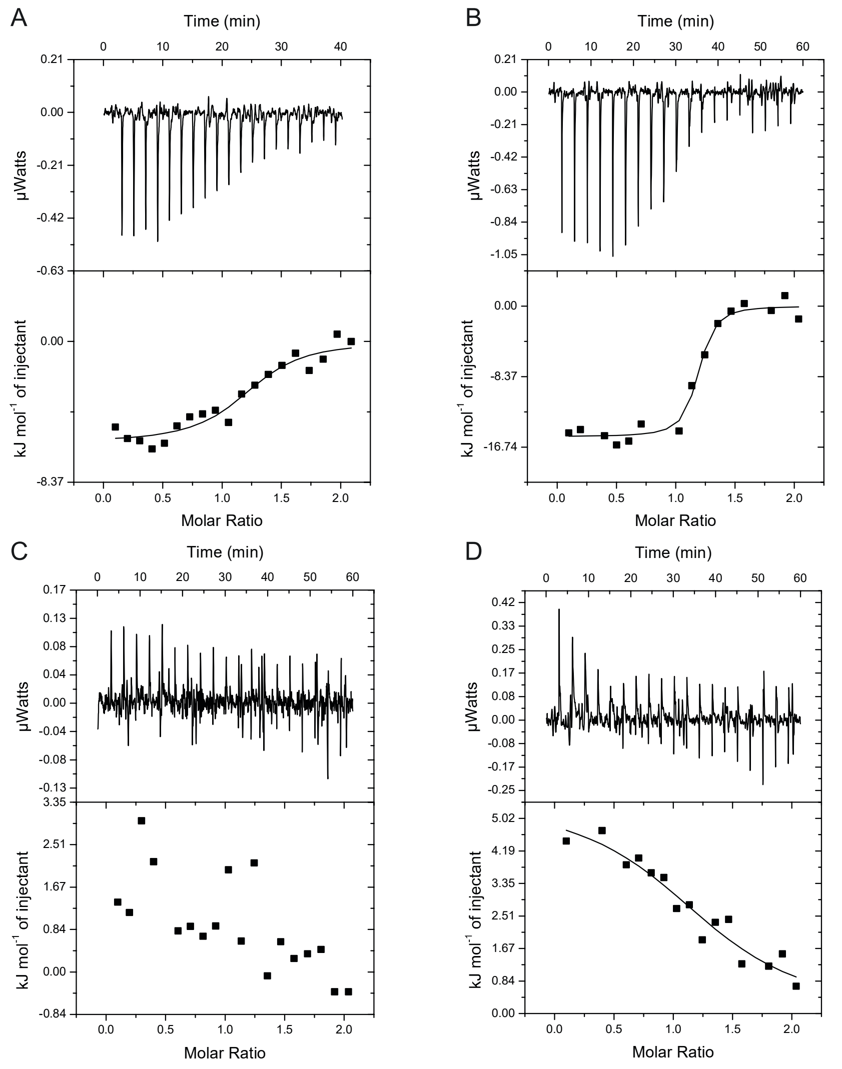

Figure S5. ITC measurements at different temperatures. ITC data showing the titration of (**A**) *At*TAF4-RST into *At*DREB2A at 25 °C, (**B**) *Hs*TAF4-TAFH into *At*DREB2A at 25 °C and (**C**) *At*TAF4-RST into ANAC013 at 25 °C and (**D**) at 10 °C. In each panel, the upper half shows baseline-corrected raw data from the titration and the lower half shows the normalized integrated binding isotherm together with the binding curve fitted to a one-site binding model.

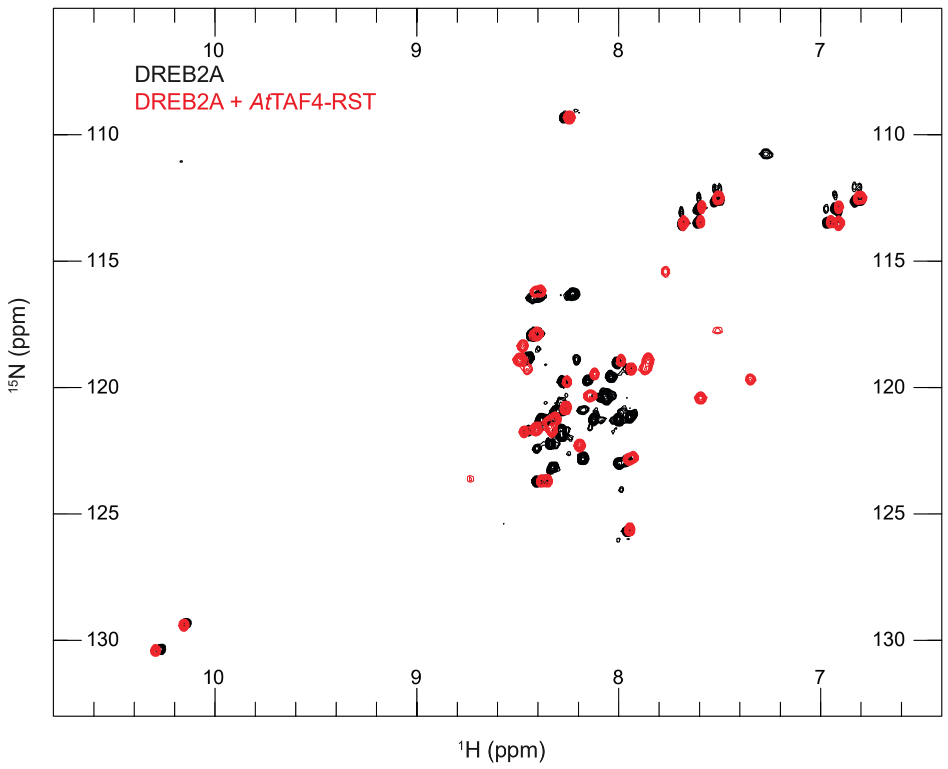

Figure S6. HSQC spectra of free and *At*TAF4-RST bound DREB2A. ^{15}N,H^{N} HSQC spectra of ^{15}N-labeled DREB2A$_{243\text{-}272}$ in the free state (black) and the *At*TAF4-RST bound state (red). Two tryptophan sidechain peaks are visible in both states around 10 ppm, this is due to the presence of a Trp-Pro motif which has an increased population of the *cis* isoform.

References

1. Staby, L., Due, A. D., Kunze, M. B. A., Jørgensen, M. L. M., Skriver, K., and Kragelund, B. B. (2021) Flanking Disorder of the Folded αα-Hub Domain from Radical Induced Cell Death1 Affects Transcription Factor Binding by Ensemble Redistribution. *Journal of Molecular Biology*. **433**, 167320
